# Fibronectin type III domain‐containing 5 improves aging‐related cardiac dysfunction in mice

**DOI:** 10.1111/acel.13556

**Published:** 2022-02-15

**Authors:** Can Hu, Xin Zhang, Min Hu, Teng Teng, Yu‐Pei Yuan, Peng Song, Chun‐Yan Kong, Si‐Chi Xu, Zhen‐Guo Ma, Qi‐Zhu Tang

**Affiliations:** ^1^ Department of Cardiology Renmin Hospital of Wuhan University Wuhan China; ^2^ Hubei Key Laboratory of Metabolic and Chronic Diseases Wuhan China

**Keywords:** aging‐related cardiac dysfunction, AMPKα, FNDC5, inflammation

## Abstract

Aging is an important risk factor for cardiovascular diseases, and aging‐related cardiac dysfunction serves as a major determinant of morbidity and mortality in elderly populations. Our previous study has identified fibronectin type III domain‐containing 5 (FNDC5) and its cleaved form, irisin, as the cardioprotectant against doxorubicin‐induced cardiomyopathy. Herein, aging or matched young mice were overexpressed with FNDC5 by adeno‐associated virus serotype 9 (AAV9) vectors, or subcutaneously infused with irisin to uncover the role of FNDC5 in aging‐related cardiac dysfunction. To verify the involvement of nucleotide‐binding oligomerization domain‐like receptor with a pyrin domain 3 (NLRP3) and AMP‐activated protein kinase α (AMPKα), *Nlrp3* or *Ampkα2* global knockout mice were used. Besides, young mice were injected with AAV9‐FNDC5 and maintained for 12 months to determine the preventive effect of FNDC5. Moreover, neonatal rat cardiomyocytes were stimulated with tumor necrosis factor‐α (TNF‐α) to examine the role of FNDC5 *in vitro*. We found that FNDC5 was downregulated in aging hearts. Cardiac‐specific overexpression of FNDC5 or irisin infusion significantly suppressed NLRP3 inflammasome and cardiac inflammation, thereby attenuating aging‐related cardiac remodeling and dysfunction. In addition, irisin treatment also inhibited cellular senescence in TNF‐α‐stimulated cardiomyocytes *in vitro*. Mechanistically, FNDC5 activated AMPKα through blocking the lysosomal degradation of glucagon‐like peptide‐1 receptor. More importantly, FNDC5 gene transfer in early life could delay the onset of cardiac dysfunction during aging process. We prove that FNDC5 improves aging‐related cardiac dysfunction by activating AMPKα, and it might be a promising therapeutic target to support cardiovascular health in elderly populations.

## Graphical abstract

FNDC5 is downregulated in aging hearts, and cardiac‐specific overexpression of FNDC5 or irisin infusion attenuates aging‐related inflammation, cardiac remodeling, and dysfunction. Mechanistically, FNDC5 activates AMPKα through blocking the lysosomal degradation of GLP‐1R.

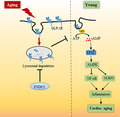

**Abbreviations**
2’5’‐dd‐Ado: 2’,5’‐dideoxyadenosine
4‐HNE: 4‐hydroxynonenal
AAV9: adeno‐associated virus serotype 9
AC: adenylyl cyclase
ACC: acetyl CoA carboxylase
AMPKα: AMP‐activated protein kinase α
Anp: atrial natriuretic peptide
ASC: apoptosis‐associated speck‐like protein
BZM: bortezomib
CFZ: carfilzomib
CHX: cycloheximide
Col1α1: collagen 1α1
DHE: dihydroethidium
Epac: exchange protein directly activated by cAMP
FNDC5: fibronectin type III domain‐containing 5
FS: fractional shortening
GLP‐1R: glucagon‐like peptide‐1 receptor
HE: hematoxylin–eosin
HW/TL: heart weight‐to‐tibia length
ItgaV: integrin αV
Itgb5: integrin β5
LVIDd: left ventricular internal dimension at end‐diastole
LVIDs: left ventricular internal dimension at end‐systole
MAP: mean arterial pressure
MDA: malondialdehyde
NF‐κB: nuclear factor‐κB
NLRP3: nucleotide‐binding oligomerization domain‐like receptor with a pyrin domain 3
PSR: picrosirius red
ROS: reactive oxygen species
SA β‐gal: Senescence‐Associated β‐Galactosidase
SNX: sorting nexins
TC: total cholesterol
TG: triglyceride
TNF‐α: tumor necrosis factor‐α
α‐Mhc: α‐myosin heavy chain

## 1 INTRODUCTION

Aging is an important risk factor for cardiovascular diseases, and aging‐related cardiac dysfunction serves as a major determinant of morbidity and mortality in elderly populations (Marian et al., 2018; Triposkiadis et al., 2019). Despite the poor understanding of all exact molecular and cellular basis, chronic cardiac inflammation has been implicated in the pathogenesis of aging‐related cardiac dysfunction through an inflammaging‐dependent manner (Liberale et al., 2020). The levels of multiple inflammatory cytokines, including interleukin‐6 (IL‐6) and tumor necrosis factor‐α (TNF‐α), are elevated in serum and heart samples of elderly populations, and closely correlate with their cardiac functional parameters and long‐term prognosis. In addition, inhibiting inflammation can provide cardiac benefits to some subjects (Gottdiener et al., 2000; Vasan et al., 2003). Nucleotide‐binding oligomerization domain‐like receptor with a pyrin domain 3 (NLRP3) inflammasome emerges as a critical sensor and regulator of inflammation, and contributes to the progression of aging‐related cardiac dysfunction (Marín Aguilar et al., 2019). Upon stimulation, apoptosis‐associated speck‐like protein (ASC) is recruited to NLRP3 scaffold to promote caspase‐1 cleavage and activation, thereby driving the maturation and release of various inflammatory mediators (e.g., IL‐1β and IL‐18) and orchestrating a pro‐inflammatory microenvironment to further amplify the inflammatory response. Therefore, it is reasonable to treat aging‐related cardiac dysfunction by inhibiting inflammation.

AMP‐activated protein kinase α (AMPKα) has pleiotropic biological functions beyond metabolic regulation and plays a critical role in controlling inflammation and cardiac homeostasis. Our previous studies have proven that AMPKα activation could alleviate inflammation and diabetes‐ or sepsis‐induced cardiac dysfunction (Ma et al., 2017; Song et al., 2020). Nuclear factor‐κB (NF‐κB) is a nodal signaling effector that provokes the transcription of various inflammatory genes, and Chen et al. revealed that AMPKα activation markedly reduced NF‐κB phosphorylation and inflammation, and subsequently prevented hypoxia/reoxygenation‐mediated cardiomyocyte injury (Chen et al., 2018). In addition, NLRP3 inflammasome pathway in the heart was also impeded by AMPKα activation (Song et al., 2020; Yang et al., 2019). AMPKα phosphorylation was found to be dampened in aging hearts, whereas activating AMPKα was sufficient to reduce aging‐induced cardiac hypertrophy and interstitial fibrosis in mice (Cieslik et al., 2017). In contrast, *Ampkα* deficiency resulted in mitochondrial damage, reactive oxygen species (ROS) overproduction, and cardiomyocyte contractile defects in aging mice (Turdi et al., 2010). These findings identify AMPKα as a promising therapeutic candidate for aging‐induced cardiac inflammation and dysfunction.

Moderate‐to‐vigorous physical activity stimulates multiple healthy benefits for elderly populations and protects against the future risk of heart failure (Kim et al., 2020). However, elderly individuals, especially those with cardiac dysfunction, are less likely to engage in and tolerate regular exercise. Inappropriate exercise also causes cardiac maladaptation and even acute cardiovascular events in susceptible people (Franklin et al., 2020). Accordingly, effective approaches mimicking the cardioprotective effects of physical exercise are of great significance for the elderly. Fibronectin type III domain‐containing 5 (FNDC5) is a type I transmembrane glycoprotein that can be proteolytically processed at the carboxy‐terminal to release irisin, an exercise‐responsive myokine conferring cardioprotection in response to different pathological stimulations, such as cardiac remodeling, ischemia/reperfusion injury, and diabetic cardiomyopathy (Bostrom et al., 2012; Zhang et al., 2020). Also, our recent study demonstrated that FNDC5 was abundant in the myocardium and that FNDC5 overexpression or irisin infusion significantly attenuated doxorubicin‐induced oxidative stress, cardiomyocyte apoptosis, and cardiac dysfunction (Zhang et al., 2020). In addition, circulating irisin levels are decreased in elderly subjects and positively correlate with the physical conditions and telomere length, suggesting a potential involvement of FNDC5 during aging process (Huh et al., 2014; Planella‐Farrugia et al., 2019). Consistently, recent studies have revealed the protective roles of FNDC5 against aging‐related memory impairment and cognitive dysfunction (Lourenco et al., 2019). In the present study, we aim to uncover the role of FNDC5 in aging‐related cardiac dysfunction and explore the underlying mechanisms.

## 2 MATERIALS AND METHODS

### 2.1 Reagents

Adeno‐associated virus serotype 9 (AAV9) vectors carrying the full‐length FNDC5 (AAV9‐FNDC5) gene under a cTnT promoter or negative control (AAV9‐NC) were generated by Hanbio Biotechnology and used in our recent study, while AAV9 vectors carrying the short hairpin RNA against glucagon‐like peptide‐1 receptor (sh*Glp*‐*1r*) or scrambled sh*RNA* were synthesized by Vigene Bioscience. Replication‐defective adenoviral vectors carrying sh*Ampkα* or scrambled sh*RNA* were purchased from Vigene Bioscience and used in our previous studies (Hu et al., 2019; Ma et al., 2016; Zhang et al., 2020). Small interfering RNAs against GLP‐1R (si*Glp*‐*1r*), exchange protein directly activated by cAMP (si*Epac*), integrin αV, integrin β5 (si*ItgaV*/*b5*), or scrambled si*RNA* were all obtained from Guangzhou RiboBio Co., Ltd. The irisin ELISA kit was purchased from Aviscera Bioscience, Inc. The assay kits to detect serum triglyceride (TG), total cholesterol (TC), malondialdehyde (MDA), or 4‐hydroxynonenal (4‐HNE) were obtained from Nanjing Jiancheng Bioengineering Institute. Senescence‐Associated β‐Galactosidase (SA β‐gal) Staining Kit was purchased from Cell Signaling Technology. The IL‐1β, IL‐6, IL‐18, and TNF‐α ELISA kits, Amplex^™^ Red Hydrogen Peroxide/Peroxidase Assay Kit, EZ‐link^™^ Sulfo‐NHS‐LC‐Biotin, and neutravidin agarose beads were purchased from Thermo Fisher Scientific. Caspase‐1 Assay Kit was purchased from Abcam. Dihydroethidium (DHE) solution was purchased from Nanjing KeyGen Biotech. Co., Ltd. Irisin, recombinant TNF‐α, lucigenin, cycloheximide (CHX, a protein synthesis inhibitor), E‐64d (a lysosomal inhibitor), leupeptin (a lysosomal inhibitor), 2’,5’‐dideoxyadenosine (2’5’‐dd‐Ado, an adenylyl cyclase/AC inhibitor), and H89 (a protein kinase A/PKA inhibitor) were purchased from Sigma‐Aldrich. Bortezomib (BZM, a reversible proteasomal inhibitor) and carfilzomib (CFZ, an irreversible proteasomal inhibitor) were purchased from Selleck Chemicals. Anti‐FNDC5 and anti‐lamin B1 were purchased from Abcam. Anti‐GAPDH, anti‐phospho‐NF‐κB p65 (p‐p65), anti‐total p65 (t‐p65), anti‐p‐AMPKα, anti‐t‐AMPKα, anti‐p‐acetyl CoA carboxylase (ACC), and anti‐t‐ACC were obtained from Cell Signaling Technology. Anti‐p16 INK4A (p16), anti‐p19 INK4D (p19), anti‐p21 Waf1 (p21), anti‐ASC, and anti‐GLP‐1R were purchased from Santa Cruz Biotechnology. Anti‐NLRP3 was purchased from Novus Biologicals, while anti‐caspase‐1 p20, anti‐ITGAV, and anti‐ITGB5 were obtained from Proteintech.

### 2.2 Animals and treatments

Male C57BL/6 mice were purchased from the Institute of Laboratory Animal Science, Chinese Academy of Medical Sciences, and housed in an environment‐controlled SPF barrier system with free access to food and water. After 1 week of adaptive feeding, 6‐month (M)‐old young and 18‐M‐old aging mice were injected with 1 × 10^11^ viral genome AAV9‐FNDC5 or AAV9‐NC per mouse from the tail vein to specifically overexpress FNDC5 in the myocardium as we previously described (Hu, Zhang, Song et al., 2020; Zhang et al., 2020). Eight weeks after AAV9 injection, mice were subjected to cardiac functional measurements and then sacrificed with the heart and serum samples collected for further investigation. To verify the role of NLRP3 and AMPKα, *Nlrp3* or *Ampkα2* global knockout (KO) mice were used according to our previous studies (Hu et al., 2019; Song et al., 2020). To enhance the clinical impact of our current work, 6‐M‐old or 18‐M‐old C57BL/6 mice were subcutaneously infused with irisin (12 nmol/kg/day) for 2 M as we previously described (Zhang et al., 2020). For the prevention study, 6‐M‐old C57BL/6 mice were intravenously injected with either AAV9‐FNDC5 or AAV9‐NC, and then maintained for an additional 12 M. To ascertain the involvement of GLP‐1R, mice were intravenously injected with 1 × 10^11^ viral genome sh*Glp*‐*1r* or sh*RNA* carried by AAV9 per mouse at 4 weeks before FNDC5 overexpression.

### 2.3 Echocardiography and hemodynamics

Echocardiography and hemodynamics were performed as we previously described (Zhang et al., 2018, Zhang, Hu, Yuan et al., 2021, Zhang, Hu, Zhang et al., 2021). Mice were placed on a preheated pad and anesthetized with 1.5% isoflurane to provide adequate sedation. Vevo^®^ 3100 High‐Resolution Preclinical Imaging System (FUJIFILM VisualSonics) was used to record functional parameters. Invasive hemodynamic parameters were collected using a 1.4F Millar catheter transducer (SPR‐839; Millar Instruments) and analyzed by the PVAN data analysis software.

### 2.4 Cell culture and treatment

Neonatal rat cardiomyocytes (NRCMs) were separated and cultured in DMEM/F12 medium containing 15% fetal bovine serum (FBS) as we previously described (Ma et al., 2019; Zhang et al., 2018, Zhang, Hu, Yuan, Yuan et al., 2021). Cells were pretreated with 20 nmol/L irisin for 24 h, followed by the stimulation with 100 ng/ml TNF‐α for an additional 24 h to mimic inflammaging *in vitro* (Cong et al., 2016; Zhang et al., 2020). To knock down endogenous AMPKα, NRCMs were pre‐infected with sh*RNA* or sh*Ampkα* (multiplicity of infection = 150) for 4 h and then maintained in fresh DMEM/F12 medium with 15% FBS for an additional 24 h before irisin treatment (Hu et al., 2019). To identify the role of GLP‐1R, EPAC, ITGAV, or ITGB5, cells were transfected with si*Glp*‐*1r*, si*Epac*, or si*ItgaV*/*b5* (50 nmol/L) using Lipo 6000^TM^ for 4 h and then cultured for an additional 24 h before irisin treatment (Hu, Zhang, Song et al., 2020, Hu, Zhang, Zhang et al., 2020). Besides, NRCMs were incubated with 2’5’‐ddAdo (200 μmol/L) or H89 (10 μmol/L) in combination with irisin for 24 h to inhibit AC or PKA, respectively (Hu, Zhang, Song et al., 2020). For *Fndc5* silence *in vitro*, NRCMs were pretransfected with si*Fndc5* (50 nmol/L) using Lipo6000^™^ for 4 h and then kept in fresh DMEM/F12 medium for an additional 24 h. To clarify FNDC5‐associated degradation of GLP‐1R, BZM (0.1 μmol/L), CFZ (1 μmol/L), E‐64d (100 nmol/L), or leupeptin (100 µmol/L) was used to inhibit proteasome‐ or lysosome‐mediated degradation, respectively (Deshotels et al., 2014; Hu et al., 2018; Jang et al., 2020; Pokorna et al., 2019). To inhibit protein synthesis, TNF‐α‐stimulated NRCMs with or without irisin incubation were treated with CHX (10 µmol/L) for indicated times (De Giusti et al., 2011).

### 2.5 Western blot and quantitative real‐time PCR

Protein extraction and Western blot were performed according to our previous studies (Hu, Zhang, Song et al., 2020; Zhang et al., 2019). Briefly, total proteins were extracted from murine hearts or cells using RIPA lysis buffer, and the concentrations were quantified with a BCA protein assay kit. Then, equal amounts of total proteins were electrophoresed by the SDS‐PAGE and transferred onto polyvinylidene fluoride membranes. After being blocked in 5% skimmed milk at room temperature for 1 h, the membranes were probed with primary antibodies at 4 °C overnight, followed by the incubation with horseradish peroxidase‐conjugated secondary antibodies at room temperature for an additional 1h on the next day. The protein bands were visualized by a ChemiDoc^™^ XRS +system and analyzed with the Image Lab software (Bio‐Rad Laboratories, Inc.). Nuclear and membrane proteins were extracted by commercial kits according to the manufacturer's instructions, and lamin B1 in nuclear fractions and GAPDH in cell lysates were used as internal controls, respectively. Total RNA was extracted using TRIzol reagent and then reversely transcribed to cDNA with a Maxima First Strand cDNA Synthesis Kit (Roche, Basel, Switzerland) (Zhang, Hu, Yuan et al., 2021, Zhang, Hu, Zhang et al., 2021). Gene expression was determined by Roche LightCycler^®^ 480 detection system using SYBR Green 1 Master Mix (Roche), and *Gapdh* was selected as an internal control.

### 2.6 Cell surface biotinylation assay

To detect the level of GLP‐1R in cell membrane, NRCMs were incubated with EZ‐link^™^ Sulfo‐NHS‐LC‐Biotin (0.5 mg/ml) at 4 °C for 30 min, followed by the quenching with 100 nmol/L Tris‐Cl (pH = 7.5) for an additional 20 min. Then, cells were lysed and incubated with neutravidin agarose beads at 4 °C overnight. The beads were subsequently washed for 4 times, and the biotin‐labeled proteins were subjected to Western blot analysis. The expression of biotin‐labeled GLP‐1R in cell surface was normalized to GAPDH in cell lysates.

### 2.7 Histological analysis

Cardiac morphology and fibrotic area were determined by hematoxylin–eosin (HE) or picrosirius red (PSR) staining as we previously described (Zhang et al., 2018, Zhang, Hu, Yuan et al., 2021, Zhang, Hu, Zhang et al., 2021). Briefly, paraffin‐embedded heart sections were dewaxed and rehydrated with dimethylbenzene and ethanol solution, and then subjected to HE or PSR staining using the standard protocols. Cardiomyocyte cross‐sectional area and interstitial collagen volume were blindly measured by two independent authors using the Image‐Pro Plus 6.0 software. Cell cross‐sectional area was averaged from 30 fields per group with at least 5 cardiomyocytes per field analyzed, and interstitial collagen volume was calculated from more than 60 fields per group. For immunohistochemistry staining, the sections were subjected to antigen retrieval with citric acid buffer, followed by the incubation with 3% hydrogen peroxide and 10% goat serum to reduce endogenous peroxidase activity or the nonspecific binding. Subsequently, the samples were probed with anti‐CD45 or anti‐CD68 at 4 °C overnight, stained with the anti‐rabbit/mouse EnVision^TM^/HRP reagent at 37 °C for 1 h, and then visualized by diaminobenzidine at room temperature (Zhang, Hu, Yuan et al., 2021, Zhang, Hu, Zhang et al., 2021). The images were captured by a light microscopy (Nikon H550L) and analyzed in a blinded manner.

### 2.8 SA β‐gal staining

SA β‐gal staining was performed to assess cell senescence *in vivo* and *in vitro* using a commercial kit according to the manufacturer's instructions. Briefly, fresh frozen heart sections or cultured NRCMs were fixed with the fixation buffer at room temperature for 15 min and then incubated with the β‐gal staining solution at 37°C for 24 h in a dry incubator. The images were captured using the light microscopy, and the percentage of SA β‐gal^+^ cells were quantified from at least 5 high‐magnification fields.

### 2.9 Telomere length measurement

Telomere length was measured based on a real‐time PCR method as previously described (Eren et al., 2014). Briefly, genomic DNA was extracted from the heart samples, and then, the ratio of telomere repeat copy number to the copy number of a single‐gene, acidic ribosomal phosphoprotein PO forward (36B4) was calculated as the telomere length.

### 2.10 Immunofluorescence staining

The abundance of GLP‐1R in cell surface was determined by immunofluorescence staining (Hu, Zhang, Zhang et al., 2020; Zhang, Hu, Yuan et al., 2021). Briefly, cell coverslips were incubated with anti‐GLP‐1R at 4 °C overnight and then probed by Alexa Fluor 568‐labeled secondary antibody at 37°C for 1 h, followed by the incubation with DAPI for nuclear visualization. The images were visualized and captured with a STELLARIS 5 confocal laser scanning microscope (Leica Microsystems Inc.).

### 2.11 Oxidative stress detection

DHE staining was performed to detect the level of superoxide anion in heart samples as we previously described (Hu et al., 2019; Zhang et al., 2020). Briefly, fresh frozen heart sections were incubated with DHE solution (5 µmol/L) at 37°C for 30 min, and then, the images were captured using a fluorescence microscope (Tokyo, Japan) in a blinded manner. Colorless and non‐fluorescent Amplex Red can be oxidized to resorufin by horseradish peroxidase in the presence of hydrogen peroxide (H_2_O_2_), a highly fluorescent product whose absorbance can be detected at 560 nm. To measure H_2_O_2_ production from the heart, fresh left ventricular blocks were incubated with Amplex Red (100 µmol/L) and horseradish peroxidase (1 U/ml) in Krebs‐HEPES buffer protected from light at 37°C for 30 min. Then, the tissue blocks were removed, and the supernatants were transferred to a 96‐well plate with the absorbance being measured at 560 nm according to the manufacturer's instructions (Griendling et al., 2016; Matsushima et al., 2013). Lucigenin undergoes one‐electron reduction to generate strong fluorescent signals that are related to the level of superoxide anion (O_2_^−^) in the sample. To detect O_2_^−^ in the heart, fresh heart samples were homogenized and incubated with 5 µmol/L lucigenin, and then, the lucigenin‐enhanced chemiluminescence was continuously measured according to previous studies (Ago et al., 2004; Matsushima et al., 2013). The levels of MDA and 4‐HNE in heart samples were detected using commercial kits according to the manufacturer's instructions as we previously described (Hu, Zhang, Song et al., 2020; Zhang et al., 2020).

### 2.12 Biochemical analysis

Fasting blood glucose (FBG) was detected using an automatic glucometer, and the levels of serum irisin, TG, and TC were measured using commercial kits according to the manufacturer's instructions. The levels of IL‐1β, IL‐6, IL‐18, and TNF‐α in cardiac extracts were determined by commercially available ELISA kits following the standard protocols. To determine caspase‐1 activity, fresh heart samples were homogenized in chilled cell lysis buffer and then incubated with YVAD‐AFC substrate (1 mmol/L) at 37 °C for 2 h in the dark, followed by the quantification at an excitation/emission wavelength of 400/505 nm. In addition, fresh heart samples were homogenized in chloroform–methanol (1:20, w:v), and then, the chloroform‐rich layer was mixed with the methanol to detect cardiac lipofuscin at an excitation/emission wavelength of 350/485 nm.

### 2.13 Statistical analysis

All values are expressed as the mean ±standard deviation and analyzed using a SPSS 22.0 software. Unpaired Student's *t* test was used to compare differences between two groups, while one‐way analysis of variance followed by Tukey's post hoc test was performed to compare the differences among multiple groups. *p* < 0.05 was considered significant.

## 3 RESULTS

### 3.1 FNDC5 attenuates aging‐related cardiac dysfunction

**FIGURE 1:**
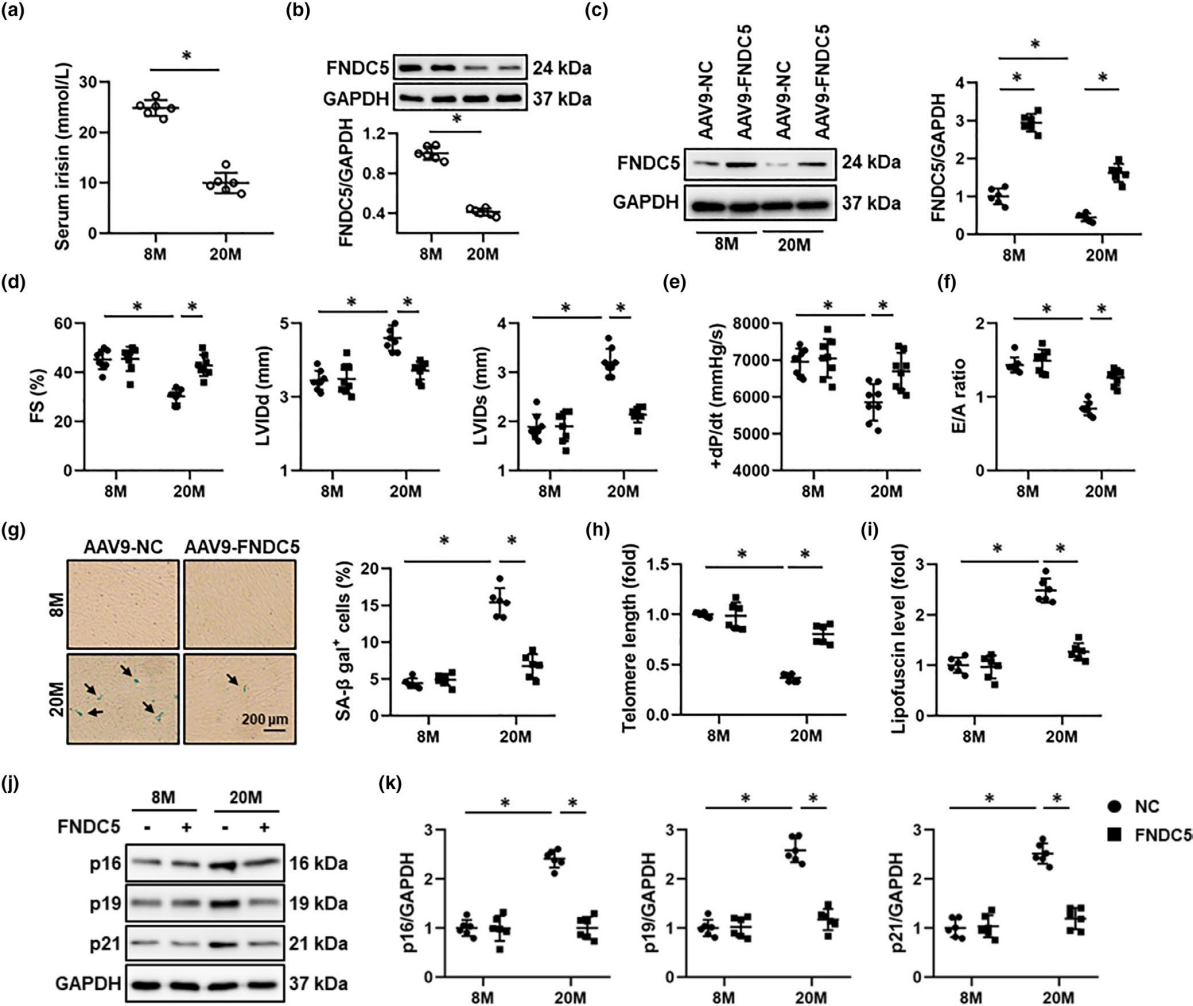
FNDC5 attenuates aging‐related cardiac dysfunction in mice. (a) The serum irisin level in 8‐month (M)‐old young and 20‐M‐old aging mice was detected by an ELISA kit (*n* = 6). (b) FNDC5 protein levels in heart samples from 8‐M‐old young and 20‐M‐old aging mice (*n* = 6). (c) 6‐M‐old young and 18‐M‐old aging mice were injected with AAV9‐FNDC5 (1 × 10^11^ viral genome per mouse) from the tail vein for 8 weeks to specifically overexpress FNDC5 in the myocardium or AAV9‐NC as a control, and then, Western blot was performed to identify the efficiency of AAV9‐FNDC5 in young (8‐M‐old) and aging (20‐M‐old) mice (*n* = 6). (d) Fractional shortening (FS), and left ventricular internal dimension at end‐diastole (LVIDd) or end‐systole (LVIDs) of mice were determined by echocardiography (*n* = 8). (e) The peak rates of isovolumic pressure development (+dP/dt) of mice (*n* = 8). (f) The ratio of the early (E) to late (A) ventricular filling velocities (*n* = 8). (g) Representative pictures of SA β‐gal‐stained heart sections and quantitative results (*n* = 6). (h) Relative telomere length in murine hearts (*n* = 6). (i) Cardiac lipofuscin content in murine hearts (*n* = 6). (j‐k) Western blot images of p16, p19, and p21, and the statistical results (*n* = 6). Values represent the mean ±standard deviation. *p < 0.05 versus the matched group

To explore the potential role of FNDC5 in aging‐related cardiac dysfunction, we firstly determined FNDC5 expression in the serum and heart of aging mice. As depicted in Figure 1a, serum irisin level was significantly decreased in aging mice. In addition, the protein and mRNA levels of FNDC5 were also reduced in aging hearts (Figure 1b and Figure S1A). Then, we specifically overexpressed FNDC5 in the myocardium with AAV9 vectors to explore the role of FNDC5 in aging‐related cardiac dysfunction. FNDC5 protein level in murine hearts was increased after AAV9 injection, whereas serum irisin abundance was not markedly affected (Figure 1c and Figure S1B). Intriguingly, FNDC5 overexpression in the heart did not affect the levels of mean arterial pressure (MAP), FBG, serum TG, and TC in aging mice (Figure S1C‐F). As shown in Figure 1d‐e, aging mice exhibited severe systolic dysfunction and ventricular dilation compared with young mice, as evidenced by the decreased fractional shortening (FS), the peak rates of isovolumic pressure development (+dP/dt) in left ventricles, and increased left ventricular internal dimension at end‐diastole (LVIDd) or end‐systole (LVIDs), which were attenuated by FNDC5 overexpression. Diastolic dysfunction is a key feature of aging hearts, and our data showed that aging mice with FNDC5 overexpression displayed improved diastolic function, as determined by the increased ratio of the early (E) to late (A) ventricular filling velocities (Figure 1f). However, no alteration of heart rate was found (Figure S1G). SA β‐gal staining was applied to identify cellular senescence in aging hearts. As shown in Figure 1g, the numbers of SA β‐gal‐positive cells in heart samples were significantly increased during aging progression, but to a less extent in those with FNDC5 overexpression. In addition, FNDC5 overexpression also preserved the telomere length in aging hearts (Figure 1h). Lipofuscin is a senescence‐associated pigment and positively correlates with the extent of aging (Marín Aguilar et al., 2019). As shown in Figure 1i, aging‐related lipofuscin accumulation in the heart was significantly suppressed by FNDC5 overexpression. Consistently, FNDC5 overexpression also reduced the protein levels of senescent markers in aging hearts, including p16, p19, and p21 (Figure 1j‐k). Collectively, our data imply that FNDC5 attenuates aging‐related cardiac dysfunction.

### 3.2 FNDC5 blocks aging‐related cardiac remodeling

**FIGURE 2:**
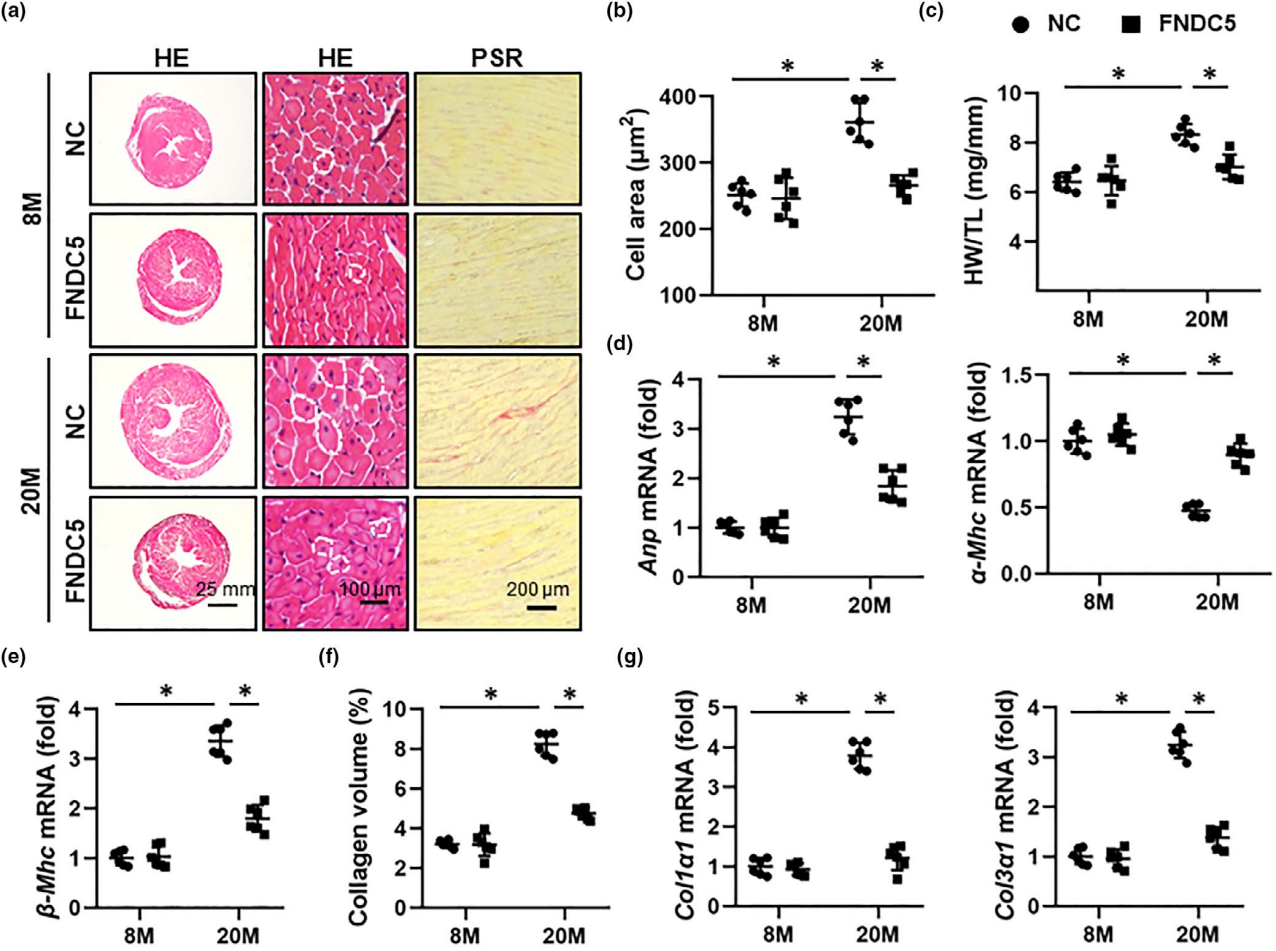
FNDC5 blocks aging‐related cardiac remodeling in mice. (a) 6‐month (M)‐old young and 18‐M‐old aging mice were injected with AAV9‐FNDC5 (1 × 10^11^ viral genome per mouse) from the tail vein for 8 weeks to specifically overexpress FNDC5 in the myocardium or AAV9‐NC as a control, and then, hematoxylin–eosin (HE) and picrosirius red (PSR) stainings were performed to evaluate cardiac remodeling (*n* = 6). (b) Quantification of the cardiomyocyte area with HE staining (*n* = 6). (c) Heart weight‐to‐tibia length (HW/TL) in mice (*n* = 6). (d‐e) Relative atrial natriuretic peptide (*Anp*), α‐myosin heavy chain (*α*‐*Mhc*), and *β*‐*Mhc* mRNA levels in murine hearts (*n* = 6). (f) Statistical results of average collagen volume by PSR staining (*n* = 6). (g) Relative collagen1α1 (*Col1α1*) and *Col3α1* mRNA levels in murine hearts (*n* = 6). Values represent the mean ±standard deviation. *p < 0.05 versus the matched group

Cardiac hypertrophy and interstitial fibrosis are key features and determinants of aging‐related cardiac dysfunction (Marian et al., 2018; Triposkiadis et al., 2019). As shown in Figure 2a‐c, aging mice displayed increased cardiac hypertrophy, as evidenced by the increased cell area and heart weight‐to‐tibia length (HW/TL), which were significantly blocked by FNDC5 overexpression. Decreased mRNA levels of hypertrophic markers, including atrial natriuretic peptide (*Anp*), α‐myosin heavy chain (*α*‐*Mhc*), and *β*‐*Mhc*, also validated the anti‐hypertrophic function of FNDC5 (Figure 2d‐e). In addition, FNDC5 overexpression dramatically suppressed collagen deposition in aging hearts, as verified by the decreased collagen volume, collagen 1α1 (*Col1α1*), and *Col3α1* mRNA levels (Figure 2f‐g). These findings indicate that FNDC5 blocks aging‐related cardiac remodeling.

### 3.3 FNDC5 suppresses aging‐associated inflammatory response

**FIGURE 3:**
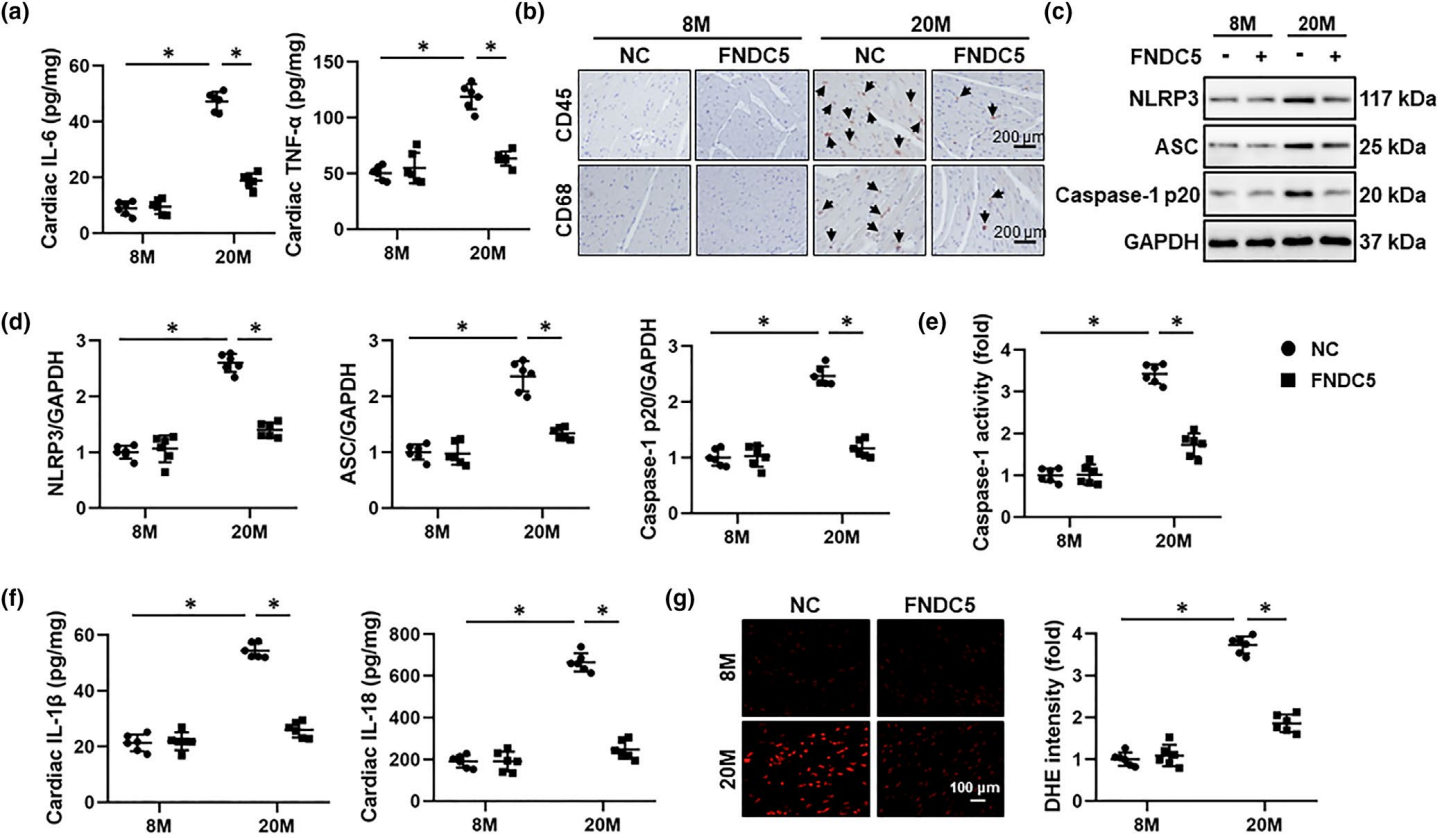
FNDC5 suppresses aging‐associated inflammatory response in mice. (a) 6‐month (M)‐old young and 18‐M‐old aging mice were injected with AAV9‐FNDC5 (1 × 10^11^ viral genome per mouse) from the tail vein for 8 weeks to specifically overexpress FNDC5 in the myocardium or AAV9‐NC as a control, and then, the myocardial interleukin‐6 (IL‐6) and tumor necrosis factor‐α (TNF‐α) levels were determined by ELISA kits (*n* = 6). (b) The immunohistochemistry staining of CD45 and CD68 in murine hearts (*n* = 6). (c‐d) Western blot images and the statistical results of p16, p19, and p21 proteins (*n* = 6). (e) The caspase‐1 activity in murine hearts (*n* = 6). (f) The myocardial IL‐1β and IL‐18 levels were determined by ELISA kits (*n* = 6). (g) Representative dihydroethidium (DHE) staining images in murine hearts and the statistical results (*n* = 6). Values represent the mean ±standard deviation. *p < 0.05 versus the matched group

Emerging evidences have addressed a direct association between low‐grade chronic inflammation and aging‐related cardiac dysfunction (Liberale et al., 2020). As shown in Figure 3a and Figure S2A, IL‐6 and TNF‐α levels were increased in aging hearts, but decreased in those with FNDC5 overexpression. Immunohistochemistry staining and PCR data identified an increased infiltration of leukocytes to murine hearts during aging progression, which were largely suppressed by FNDC5 overexpression (Figure 3b and Figure S2B). NF‐κB is a nodal signaling effector in orchestrating the transcription of various inflammatory genes. As expected, FNDC5 overexpression remarkably reduced the phosphorylation and nuclear translocation of NF‐κB p65 (Figure S2C‐D). NLRP3 inflammasome emerges as a critical sensor and regulator of inflammation, and contributes to the progression of aging‐related cardiac dysfunction (Marín Aguilar et al., 2019). As shown in Figure 3c‐d, aging‐related upregulation of NLRP3, ASC, and cleaved caspase‐1 p20 was decreased by FNDC5. Meanwhile, FNDC5 overexpression also suppressed cardiac caspase‐1 activity, accompanied by decreased IL‐1β and IL‐18 levels in aging hearts (Figure 3e‐f). ROS overproduction is involved in the activation of NLRP3 inflammasome, and our recent study has determined an antioxidant capacity of FNDC5 in murine hearts; therefore, we sought to investigate whether FNDC5 overexpression could mitigate aging‐related oxidative stress (Song et al., 2020; Zhang et al., 2020). As shown in Figure 3g, DHE staining data revealed that FNDC5 overexpression significantly reduced aging‐related oxidative stress in murine hearts. In addition, the productions of H_2_O_2_ and O_2_^−^ in aging hearts were also evidently suppressed with FNDC5 overexpression, accompanied by decreased cardiac 4‐HNE and MDA levels (Figure S2E‐F). To further validate the involvement of NLRP3 inflammasome in FNDC5‐mediated cardioprotection against aging, *Nlrp3* global KO mice were used. As shown in Figure S3A‐B, FNDC5 overexpression significantly reduced cardiac IL‐6, TNF‐α, IL‐1β, and IL‐18 levels in aging mice, yet failed to do so in *Nlrp3* KO aging mice. Also, FNDC5 failed to improve aging‐related systolic and diastolic dysfunction in *Nlrp3*‐deficient mice, as evidenced by the unaltered FS, +dP/dt, and E/A ratio (Figure S3C‐D). In addition, the protective effects of FNDC5 on telomere length and cardiac lipofuscin accumulation were abrogated after the knockout of NLRP3 (Figure S3E‐F). Meanwhile, FNDC5 lost the anti‐hypertrophic and anti‐fibrotic roles in aging mice with *Nlrp3* deficiency (Figure S3G‐H). These findings suggest that inhibiting NLRP3 inflammasome is required for FNDC5‐mediated cardioprotective effects against aging. To further validate the role of FNDC5 against inflammation‐associated cardiac aging, TNF‐α, a critical inflammatory cytokine in aging hearts, was used to treat NRCMs to mimic inflammaging process *in vitro* according to a previous study (Cong et al., 2016). As shown in Figure S4A, irisin treatment dramatically reduced SA β‐gal‐positive cells upon TNF‐α stimulation. Also, TNF‐α‐induced increases in senescent markers were also decreased by irisin incubation (Figure S4B‐C). Our results validate that FNDC5 suppresses aging‐associated inflammatory response.

### 3.4 FNDC5 protects against aging‐related cardiac dysfunction by activating AMPKα

**FIGURE 4:**
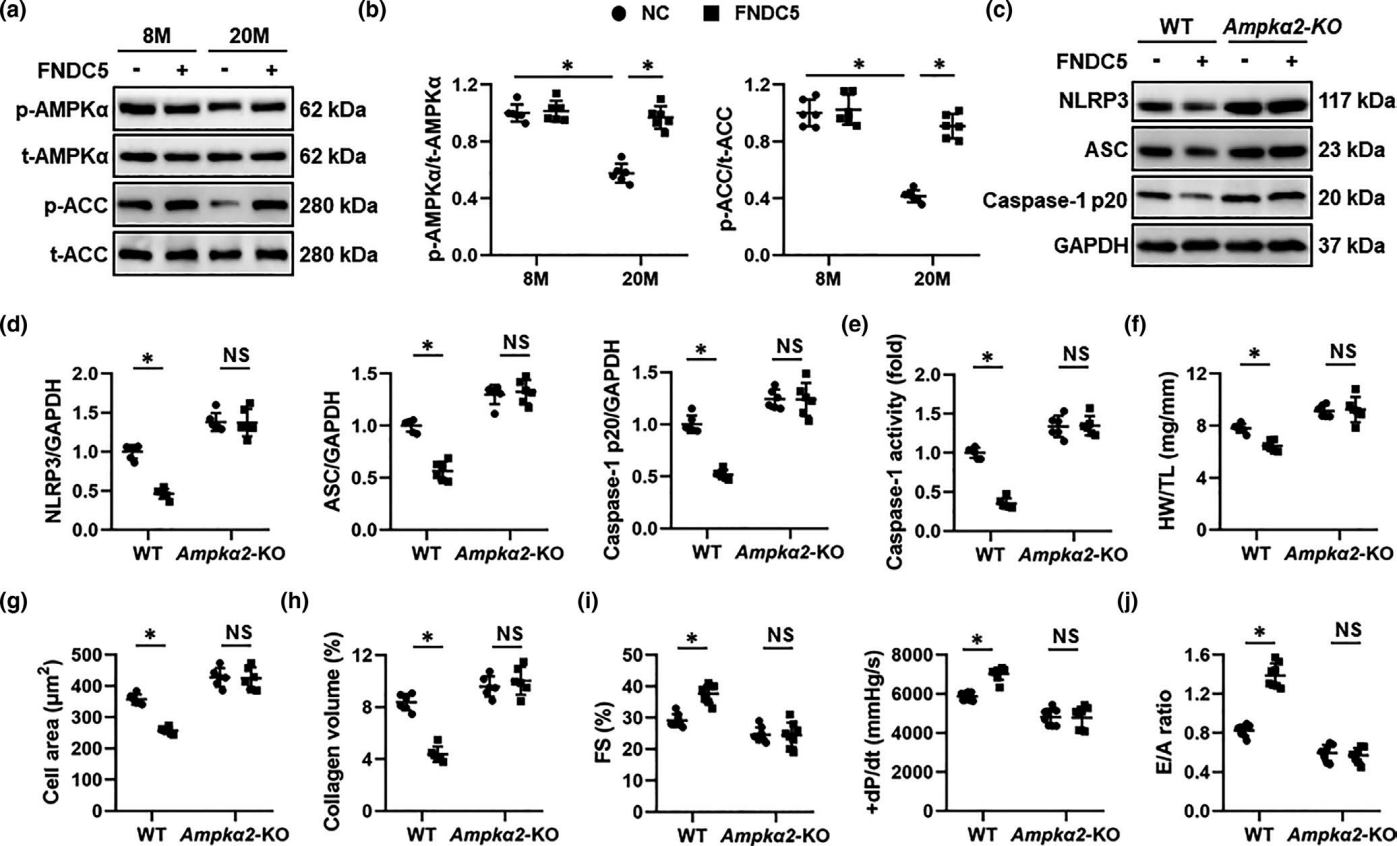
FNDC5 protects against aging‐related cardiac dysfunction by activating AMPKα. (a‐b) 6‐month (M)‐old young and 18‐M‐old aging mice were injected with AAV9‐FNDC5 (1 × 10^11^ viral genome per mouse) from the tail vein for 8 weeks to specifically overexpress FNDC5 in the myocardium or AAV9‐NC as a control, and then, Western blot was performed to detect AMPKα and downstream acetyl CoA carboxylase (ACC) phosphorylation in murine hearts (*n* = 6). (c‐d) Western blot images and the statistical results of nucleotide‐binding oligomerization domain‐like receptor with a pyrin domain 3 (NLRP3), apoptosis‐associated speck‐like protein (ASC), and caspase‐1 p20 proteins (*n* = 6). (e) The caspase‐1 activity in murine hearts (*n* = 6). (f) Heart weight‐to‐tibia length (HW/TL) in mice (*n* = 6). (g) Quantification of the cardiomyocyte area in mice (*n* = 6). (h) Average collagen volume in mice (*n* = 6). (i) Echocardiographic and hemodynamic parameters of cardiac function in mice, including fractional shortening (FS) and the peak rates of isovolumic pressure development (+dP/dt) in mice (*n* = 8). (j) The ratio of the early (E) to late (A) ventricular filling velocities (*n* = 8). Values represent the mean ±standard deviation. *p < 0.05 versus the matched group. NS indicates no significance

AMPKα is a longevity‐related molecule and plays critical roles in controlling inflammation and cardiac homeostasis (Salminen, & Kaarniranta, 2012). Our previous studies have proven that AMPKα activation could alleviate inflammation and diabetes‐ or sepsis‐induced cardiac dysfunction (Ma et al., 2017; Song et al., 2020). Herein, we found that FNDC5 overexpression significantly elevated AMPKα activity in aging hearts, as determined by the increased phosphorylation of AMPKα and the downstream ACC (Figure 4a‐b). To gain the evidence that AMPKα activation was responsible for the anti‐inflammatory and cardioprotective effects of FNDC5 in aging mice, *Ampkα2* global KO mice were used. As shown in Figure 4c‐e, *Ampkα* deficiency completely abolished FNDC5 overexpression‐mediated suppression on NLRP3 inflammasome. In addition, FNDC5 overexpression also failed to inhibit NF‐κB activation in *Ampkα*‐deficient aging hearts (Figure S5A‐B). Meanwhile, the decreased IL‐6 and TNF‐α levels seen in FNDC5‐overexpressed aging hearts were dramatically blunted in *Ampkα2* KO mice (Figure S5C). As shown in Figure S5D‐E, FNDC5 significantly preserved the telomere length and reduced cardiac lipofuscin accumulation in aging hearts, yet failed to do so in *Ampkα2* KO mice. Accordingly, FNDC5 lost its inhibitory effects on p16, p19, and p21 expressions in *Ampkα*‐deficient aging hearts (Figure S5F‐G). Also, *Ampkα* deficiency also blocked FNDC5 overexpression‐mediated suppressions on cardiac hypertrophy and fibrosis in aging mice (Figure 4f‐h). As expected, the improved systolic and diastolic function in aging hearts with FNDC5 overexpression was evidently negated by *Ampkα* deficiency (Figure 4i‐j). To further validate the role of AMPKα, NRCMs were pretreated with sh*Ampkα* to deplete AMPKα *in vitro*. Consistent with the data *in vivo*, we observed that the inhibitory effect of irisin on cellular senescence in TNF‐α‐treated NRCMs was abolished by *Ampkα* deletion (Figure S6A‐C). Taken together, we demonstrate that FNDC5 protects against aging‐related cardiac dysfunction by activating AMPKα.

### 3.5 FNDC5 activates AMPKα via blocking the lysosomal degradation of GLP‐1R

**FIGURE 5:**
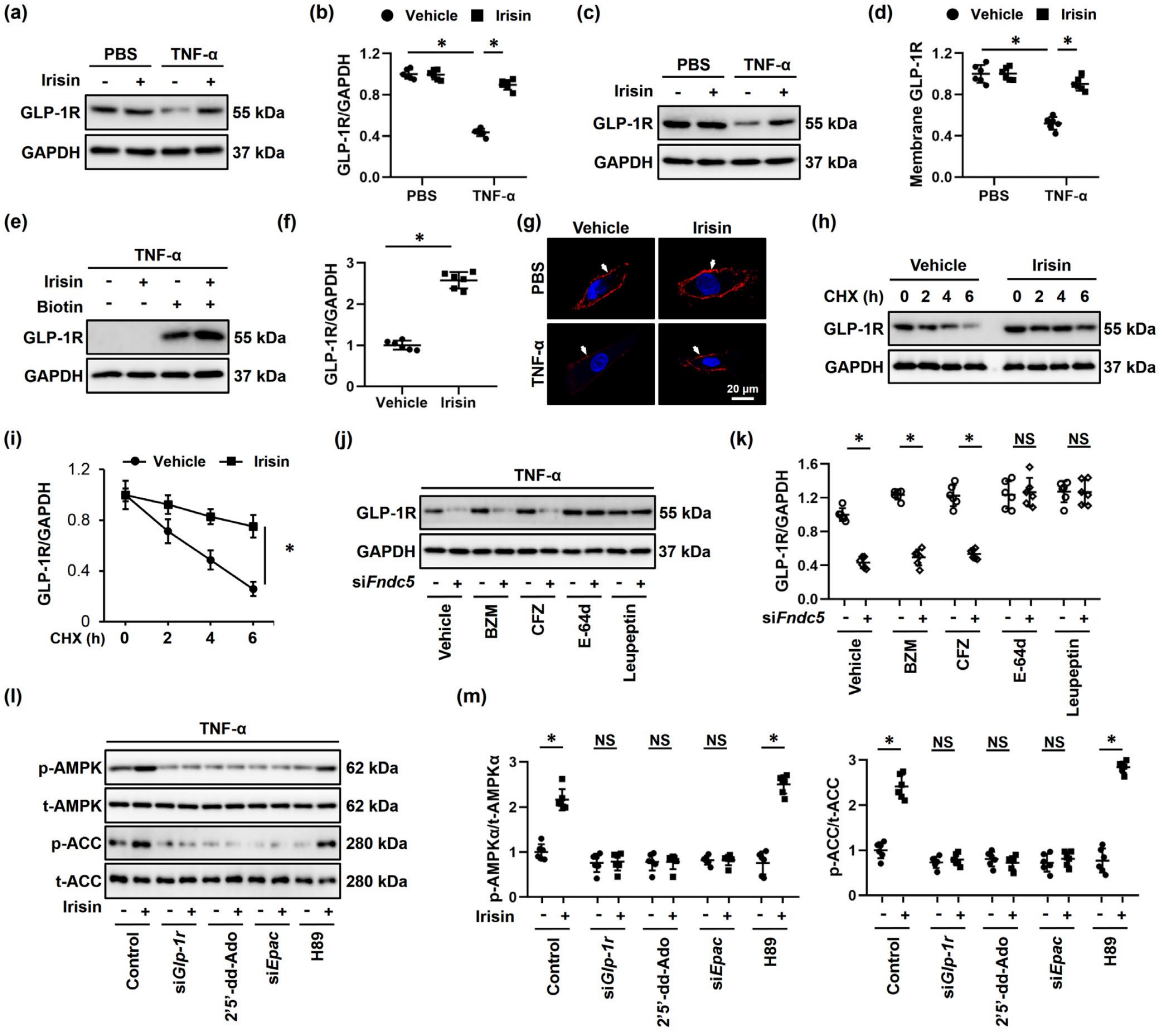
FNDC5 activates AMPKα via blocking the lysosomal degradation of GLP‐1R in vitro. (a‐d) Neonatal rat cardiomyocytes (NRCMs) were treated with 20 nmol/L irisin for 24 h, followed by the stimulation with 100 ng/ml tumor necrosis factor‐α (TNF‐α) or phosphate‐buffered saline (PBS) for an additional 24 h to mimic inflammaging *in vitro*, and GLP‐1R expressions in total cell lysates or membrane fractions were determined by Western blot (*n* = 6). (e‐f) NRCMs were subjected to cell surface biotinylation assay, and biotin‐labeled GLP‐1R expression in cell surface was determined by Western blot and normalized to GAPDH in cell lysates. (*n* = 6). (g) Immunofluorescence staining of GLP‐1R to determine the membrane localization in NRCMs (*n* = 6). (h‐i) Cells were pretreated with 20 nmol/L irisin for 24 h, followed by the stimulation with 100 ng/ml TNF‐α for an additional 24 h to mimic inflammaging in vitro, and then, cycloheximide (CHX, 10 µmol/L) was used to inhibit protein synthesis at indicated times. GLP‐1R expression in cell lysates was determined by Western blot (*n* = 6). (j‐k) NRCMs were pretransfected with si*Fndc5* (50 nmol/L) for 4 h and then maintained for an additional 24 h. To clarify FNDC5‐associated degradation of GLP‐1R, bortezomib (BZM, 0.1 μmol/L), carfilzomib (CFZ, 1 μmol/L), E‐64d (100 nmol/L), or leupeptin (100 µmol/L) was used to inhibit proteasome‐ or lysosome‐mediated degradation. Also, GLP‐1R expression in cell lysates was determined by Western blot. (l‐m) NRCMs were treated with 20 nmol/L irisin for 24 h and then stimulated with 100 ng/ml tumor necrosis factor‐α (TNF‐α) for an additional 24 h. To suppress GLP‐1R, adenylyl cyclase (AC), exchange protein directly activated by cAMP (EPAC), or protein kinase A (PKA), cells were treated with si*Glp*‐*1r* (50 nmol/L), 2’,5’‐dideoxyadenosine (2’5’‐dd‐Ado, 200 μmol/L), si*Epac* (50 nmol/L), or H89 (10 μmol/L). Subsequently, AMPKα and downstream acetyl CoA carboxylase (ACC) phosphorylation in murine hearts were determined (*n* = 6). Values represent the mean ±standard deviation. *p < 0.05 versus the matched group. NS indicates no significance

The members of integrin family, especially ITGAV and ITGB5, are potential receptors of irisin in osteocytes and fat cells, and simultaneous knockdown of ITGAV and ITGB5 also blocked FNDC5‐mediated protective effects against diabetic cardiomyopathy (Kim et al., 2019; Lin et al., 2021). Intriguingly, AMPKα activation and anti‐aging effects by irisin upon TNF‐α stimulation were not affected by *ItgaV*/*b5* silence (Figure S7A‐E). We previously reported that GLP‐1R upregulation was sufficient to activate AMPKα pathway and that *Glp*‐*1r* silence significantly restrained AMPKα activation in the heart (Ma et al., 2016; Song et al., 2020). Also, Gros et al. determined that *Glp*‐*1r*‐deficient mice exhibited elevated left ventricular end‐diastolic pressure and ventricular thickness at baseline, and displayed systolic and diastolic impairment upon insulin administration (Gros et al., 2003). Based on these findings, we tried to explore whether FNDC5 activated AMPKα through GLP‐1R. As shown in Figure 5a‐b, irisin treatment significantly preserved GLP‐1R expression in TNF‐α‐treated NRCMs. Moreover, the membrane localization of GLP‐1R in TNF‐α‐treated NRCMs was also increased in the presence of irisin (Figure 5c‐g). Notably, neither irisin treatment nor FNDC5 overexpression altered *Glp*‐*1r* mRNA levels *in vitro* and *in vivo*; however, GLP‐1R degradation in NRCMs was reduced by irisin treatment (Figure S8A and Figure 5h‐i). To further clarify the role of endogenous FNDC5 on GLP‐1R expression *in vitro*, we knocked down the expression of FNDC5 in TNF‐α‐treated NRCMs using si*Fndc5*. As expected, *Fndc5* silence significantly accelerated GLP‐1R degradation upon TNF‐α stimulation (Figure 5j‐k). GLP‐1R is degraded through both proteasomal and lysosomal pathways after internalization (Jones et al., 2018). Therefore, we treated NRCMs with either proteasomal inhibitors (BZM and CFZ) or lysosomal inhibitors (E‐64d and leupeptin). As shown in Figure 5j‐k, GLP‐1R degradation in TNF‐α‐treated *Fndc5*‐deficient NRCMs was prevented by both E‐64d and leupeptin, instead of BZM or CFZ, indicating that the increased expression and membrane localization of GLP‐1R in irisin‐treated NRCMs were probably secondary to the inhibition of its lysosomal degradation. We previously showed that GLP‐1R activation triggered AMPKα pathway through the cAMP/EPAC axis (Song et al., 2020). Consistently, AMPKα activation in irisin‐treated NRCMs was blocked by si*Glp*‐*1r*, 2’5’‐dd‐Ado or si*Epac*, but not H89 (Figure 5l‐m). As expected, irisin‐induced inhibitions on p16, p19, and p21 were completely offset in *Glp*‐*1r*‐deficient NRCMs (Figure S8C‐F). To further verify the necessity of GLP‐1R in FNDC5 overexpression‐mediated AMPKα activation and cardioprotection, aging mice were pre‐injected with sh*Glp*‐*1r* to knock down endogenous GLP‐1R expression in the heart (Figure S9A). As shown in Figure S9B‐C, AMPKα activation by FNDC5 was absolutely blunted by *Glp*‐*1r* silence in aging hearts. Accordingly, *Glp*‐*1r* silence significantly blocked FNDC5 overexpression‐mediated protective effects on cardiac remodeling and dysfunction (Figure S9D‐I). Also, the decreased protein levels of p16, p19, and p21 in aging hearts by FNDC5 overexpression were also abrogated in the absence of GLP‐1R (Figure S9J‐K). Together, we prove that FNDC5 activates AMPKα by blocking the lysosomal degradation of GLP‐1R.

### 3.6 Irisin infusion mitigates aging‐related cardiac dysfunction

**FIGURE 6:**
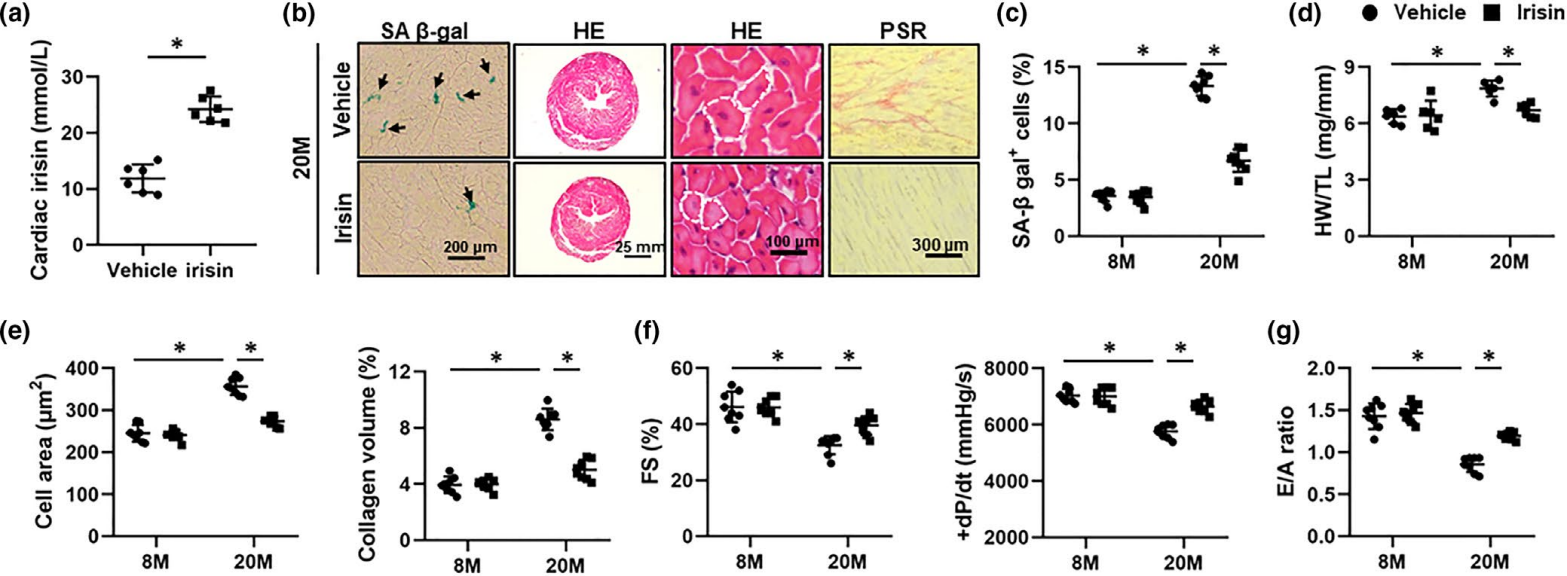
Irisin infusion mitigates aging‐related cardiac dysfunction in mice. (a) 6‐month (M)‐old or 18‐M‐old mice were subcutaneously infused with irisin (12 nmol/kg/day) for 2 M, and the irisin level in aging hearts was detected by an ELISA kit. (*n* = 6). (b) Representative images of SA β‐gal, hematoxylin–eosin (HE) and picric sirius red (PSR) staining in aging hearts (*n* = 6). (c) Quantitative data of SA β‐gal‐positive cells in heart tissues (*n* = 8). (d) Heart weight‐to‐tibia length (HW/TL) (*n* = 6). (e) Quantification of cardiomyocyte area and average collagen volume in mice (*n* = 8). (f) Echocardiographic and hemodynamic parameters of cardiac function in mice, including fractional shortening (FS) and the peak rates of isovolumic pressure development (+dP/dt) in mice (*n* = 8). (g) The ratio of the early (E) to late (A) ventricular filling velocities (*n* = 8). Values represent the mean ±standard deviation. *p < 0.05 versus the matched group

We next investigated whether infusion of exogenous irisin would mitigate aging‐related cardiac dysfunction in mice (Figure 6a). As shown in Figure 6b‐c, irisin infusion for 2 M significantly reduced the numbers of SA β‐gal‐positive cells in aging hearts. Also, aging‐induced cardiac hypertrophy and fibrosis were also suppressed by irisin treatment (Figure 6d‐e). Consistent with the phenotypic alterations, irisin infusion dramatically decreased the protein levels of senescent markers, including p16, p19, and p21 (Figure S10A‐B). As expected, aging‐related systolic and diastolic impairment was also improved by irisin infusion, as evidenced by the increased FS, +dP/dt, and E/A ratio (Figure 6f‐g). All these data reveal that irisin infusion mitigates aging‐related cardiac dysfunction.

### 3.7 FNDC5 delays the onset of cardiac dysfunction during aging process

Given the cardioprotective role of FNDC5 in aging mice, we sought to determine whether FNDC5 overexpression in early life would delay the onset of cardiac dysfunction during aging process. As shown in Figure S11A‐B, FNDC5‐overexpressed mice displayed improved cardiac function during aging process. In addition, the protein levels of p16, p19, and p21 were also reduced in aging hearts with FNDC5 overexpression (Figure S11C‐D). Meanwhile, FNDC5 overexpression in early life significantly reduced the numbers of SA β‐gal‐positive cells and alleviated cardiac hypertrophy and fibrosis in aging hearts (Figure S11E‐H). In conclusion, we verify that FNDC5 delays the onset of cardiac dysfunction during aging process.

## 4 DISCUSSION

Epidemiological studies indicate that cardiovascular diseases are leading causes of death in the elderly and that aging‐related cardiac dysfunction imposes a major financial burden on the whole society due to the extended average life span. In the present study, we test the feasibility of FNDC5 on aging‐related cardiac dysfunction in both treatment and prevention strategies. We demonstrate that cardiac‐specific overexpression of FNDC5 or irisin treatment significantly suppresses cardiac inflammation, thereby alleviating cellular senescence and cardiac dysfunction in aging mice. Mechanistically, we prove that FNDC5 activates AMPKα through blocking the lysosomal degradation of GLP‐1R. More importantly, we employ a prevention study and determine that FNDC5 gene transfer in early life could delay the onset of cardiac dysfunction during aging process. Therefore, our present study identifies FNDC5 as a promising therapeutic target to support cardiovascular health in elderly populations.

Aging‐related cardiac dysfunction exhibits distinctive epidemiological, histological, and biological features after the long‐term exposure to various risk factors and intrinsic aging disabilities (Triposkiadis et al., 2019). It has been reported that aging hearts usually develop increased left ventricular wall thickness, cardiomyocyte hypertrophy, and interstitial collagen deposition, which in turn result in systolic and diastolic dysfunction due to the increased ventricular wall stiffness, decreased compliance, and impaired ventricular filling (Marian et al., 2018; Triposkiadis et al., 2019). In the current study, we demonstrated that FNDC5 overexpression significantly attenuated cardiac remodeling, and systolic and diastolic dysfunction in aging hearts. The chronic inflammatory status is reported as a characteristic feature of aging‐related physical disorders (Liberale et al., 2020). Various inflammatory biomarkers, including IL‐6 and TNF‐α, are identified as predictors of many aging phenotypes (e.g., energy metabolism, immune senescence, and muscle degeneration) (Furman et al., 2017). Chronic inflammation inherent to aging process may contribute to the development of immune intolerance in the elderly, which negatively impacts the response to potential danger and thus increases the susceptibility to cardiac dysfunction of aging populations (Liberale et al., 2020; Marín Aguilar et al., 2019). NLRP3 inflammasome is essential for the cleavage and maturation of various inflammatory cytokines, and triggers the rapid release of IL‐1β and IL‐18 to construct a pro‐inflammatory microenvironment, which in turn recruit macrophages and other leukocytes to further amplify the inflammatory response. Consistently, Marín et al. previously demonstrated that *Nlrp3* deficiency preserved cardiac function and life span in aging mice (Marín Aguilar et al., 2019). In addition, genetic inactivation of NLRP3 also significantly prevented the activation of fetal gene program and partially improved cardiac impairment of aging hearts after β‐agonistic stimulation (Marneros, 2018). Although the exact mechanisms involved in the activation of NLRP3 inflammasome are poorly understood, ROS overproduction is sufficient to activate NLRP3 inflammasome through inducing removal of thioredoxin‐interacting protein from thioredoxin to NLRP3. We previously reported that FNDC5 overexpression could reduce ROS generation in the heart upon doxorubicin treatment. Herein, FNDC5 overexpression also attenuated aging‐related oxidative stress, accompanied by suppressions of NLRP3 inflammasome, and decreased cardiac inflammation. More importantly, we verify that FNDC5 lost its cardioprotection against aging in *Nlrp3*‐deficient mice. Our data propose FNDC5 as a promising therapeutic target to treat aging‐related cardiac dysfunction.

As a central metabolic biosensor during aging process, AMPKα is increasingly recognized as a potential longevity‐related molecule with anti‐inflammatory properties (Salminen, & Kaarniranta, 2012). In this study, we also validate that FNDC5 overexpression improves aging‐related inflammation and cardiac dysfunction by activating AMPKα. Despite the verification of a relationship between FNDC5 and AMPKα in many studies, relatively little is known about how FNDC5 activates AMPKα. GLP‐1R is a member of the G protein‐coupled receptors that transduces extracellular signals to intracellular molecular network through AC/cAMP axis. Emerging studies have indicated that GLP‐1R is expressed in multiple tissues, including the heart (Helmstadter et al., 2021). Also, Gros et al. also determined the necessity of GLP‐1R in aging‐related cardiac remodeling and dysfunction. They showed that *Glp*‐*1r*‐deficient mice exhibited elevated left ventricular end‐diastolic pressure and ventricular thickness at baseline, and displayed systolic and diastolic impairment upon insulin administration (Gros et al., 2003). In addition, our previous studies demonstrated that GLP‐1R activation was sufficient to attenuate pressure overload‐, obesity‐, and sepsis‐induced cardiac dysfunction. Currently, various GLP‐1R agonists approved for the treatment of diabetes also stimulate cardiovascular benefits in some populations (Helmstadter et al., 2021). However, agonist‐mediated endocytosis can provoke the degradation of GLP‐1R in some cases, which extremely impedes the clinical application of GLP‐1R agonists (Jones et al., 2018). In the present study, we proved that FNDC5 significantly suppressed the lysosomal degradation of GLP‐1R, thereby elevating GLP‐1R expression and membrane localization. The degradation of GLP‐1R commonly involves clathrin‐ or caveolin‐dependent endocytic machinery, spatiotemporal sorting, intracellular degradation, and recycling back to the plasma membrane (Jones et al., 2018; Sonoda et al., 2008; Wootten et al., 2016). Yet, the exact mechanisms through which FNDC5 regulates the lysosomal degradation and net surface expression of GLP‐1R remain elusive in this study. GLP‐1R degradation in most cases depends on the recruitment of clathrin or caveolin adaptors to promote endocytosis and desensitization; however, GLP‐1R internalization is also required for signal transduction of some GLP‐1R agonists (e.g., exendin‐4). Therefore, completely blocking GLP‐1R endocytosis may suppress downstream signaling pathways (Widmann et al., 1995). After internalization, GLP‐1R is differentially sorted into proteasomes or lysosomes with the help of sorting nexins (SNX), where it is degraded by ubiquitin–proteasome proteolytic pathway or lysosomal enzymes. In addition, Buenaventura et al. previously determined that SNX27 and SNX1 were essential for controlling the balance between GLP‐1R lysosomal degradation and plasma membrane recycling (Buenaventura et al., 2018). Lysosomes are well accepted as a major post‐endocytic GLP‐1R destination, and our present data consistently reveal that FNDC5 downregulation upon inflammaging stimulation accelerates the lysosomal degradation of GLP‐1R and decreases its membrane localization, thereby inhibiting downstream AMPKα signaling and cardioprotection. In addition, the net surface GLP‐1R expression also depends on GLP‐1R membrane recycling (Widmann et al., 1995). Although we prove that FNDC5 suppresses GLP‐1R lysosomal degradation and subsequently activates downstream AMPKα pathway, whether other mechanisms exist to increase its net surface abundance demands further investigation.

In summary, we prove that FNDC5 improves aging‐related cardiac dysfunction by activating AMPKα, and it might be a promising therapeutic target to support cardiovascular health in elderly populations.

## CONFLICT OF INTEREST

The authors declare no conflicts of interest.

## AUTHOR CONTRIBUTIONS

Can Hu, Xin Zhang, and Qi‐Zhu Tang conceived the project. Can Hu, Xin Zhang, Zhen‐Guo Ma, and Qi‐Zhu Tang designed the experiments. Can Hu, Xin Zhang, Min Hu, Teng Teng, Yu‐Pei Yuan, and Si‐Chi Xu performed the study and acquired data. Can Hu, Xin Zhang, Min Hu, Peng Song, and Chun‐Yan Kong conducted data analysis and contributed to the data interpretation. Can Hu and Xin Zhang drafted and revised the manuscript. Min Hu helped to revise the manuscript. Zhen‐Guo Ma and Qi‐Zhu Tang were responsible for the financial support, study supervision, and final approval of the manuscript.

## Supporting information

Supplementary Material

## Data Availability

All data that support the findings of this study are available from the corresponding author upon reasonable request.
