# Supplementary material for: Fibronectin type III domain‐containing 5 improves aging‐related cardiac dysfunction in mice
Source: Aging Cell. 2022 Feb 15;21(3):e13556. doi: 10.1111/acel.13556 (PMC8920441; doi:10.1111/acel.13556)
Supplement: Supplementary file 1 — Supplementary Material [file ACEL-21-e13556-s001.docx]

**Supporting information**

**Fibronectin type III domain-containing 5 improves aging-related cardiac dysfunction in mice**

Can Hu^1, 2^ ^*^, Xin Zhang^1, 2^ ^*^, Min Hu^1, 2^, Teng Teng^1, 2^, Yu-Pei Yuan^1, 2^, Peng Song^1, 2^,

Chun-Yan Kong^1, 2^, Si-Chi Xu^1, 2^, Zhen-Guo Ma^1, 2^ & Qi-Zhu Tang^1, 2^

^1^ Department of Cardiology, Renmin Hospital of Wuhan University, Wuhan 430060, China

^2^ Hubei Key Laboratory of Metabolic and Chronic Diseases, Wuhan 430060, China

^*^ These authors contributed equally to this work.

Corresponding author:

**Qi-Zhu Tang**,

Department of Cardiology,

Renmin Hospital of Wuhan University,

Hubei Key Laboratory of Metabolic and Chronic Diseases,

Wuhan University at Jiefang Road 238, Wuhan 430060, PR China

Tel.: +86 027-88073385; Fax: +86 027-88042292.

E-mail: [qztang@whu.edu.cn](mailto:qztang@whu.edu.cn) (Qi-Zhu Tang).

**Supplementary Table: primer sequences used for quantitative real-time PCR**

| **Gene** | **Species** |  | **Sequence** |
| --- | --- | --- | --- |
| *Gapdh* | Mouse | Forward | ACTCCACTCACGGCAAATTC |
|  |  | reverse | TCTCCATGGTGGTGAAGACA |
| *Anp* | Mouse | Forward | ACCTGCTAGACCACCTGGAG |
|  |  | reverse | CCTTGGCTGTTATCTTCGGTACCGG |
| *α-Mhc* | Mouse | Forward | GGATGCCCTGCTGGTTA |
|  |  | reverse | CGCCCAAACTCCTCCTT |
| *β-Mhc* | Mouse | Forward | CCGAGTCCCAGGTCAACAA |
|  |  | reverse | CTTCACGGGCACCCTTGGA |
| *Col1α1* | Mouse | Forward | AGGCTTCAGTGGT T TGGATG |
|  |  | reverse | CACCAACAGCACCATCGTTA |
| *Col3α1* | Mouse | Forward | CCCAACCCAGAGATCCCATT |
|  |  | reverse | GAAGCACAGGAGCAGGTGTAGA |
| *Il-6* | Mouse | Forward | AGTTGCCTTCTTGGGACTGA |
|  |  | reverse | TCCACGATTTCCCAGAGAAC |
| *Tnf-α* | Mouse | Forward | GCAAAGGGAGAGTGGTCA |
|  |  | reverse | CTGGCTCTGTGAGGAAGG |
| *Glp-1r* | Mouse | Forward | GCCTTTGTGATGGACGAAC |
|  |  | reverse | GCCCTGGAAGGAAGTGAA |
| *Cd45* | Mouse | Forward | GGTGACTTTTGGCAGATGA |
|  |  | reverse | TTTCCTTCGCCCCAGTA |
| *Cd68* | Mouse | Forward | TGTCTGATCTTGCTAGGACCG |
|  |  | reverse | GAGAGTAACGGCCTTTTTGTGA |

**Supplementary figures**


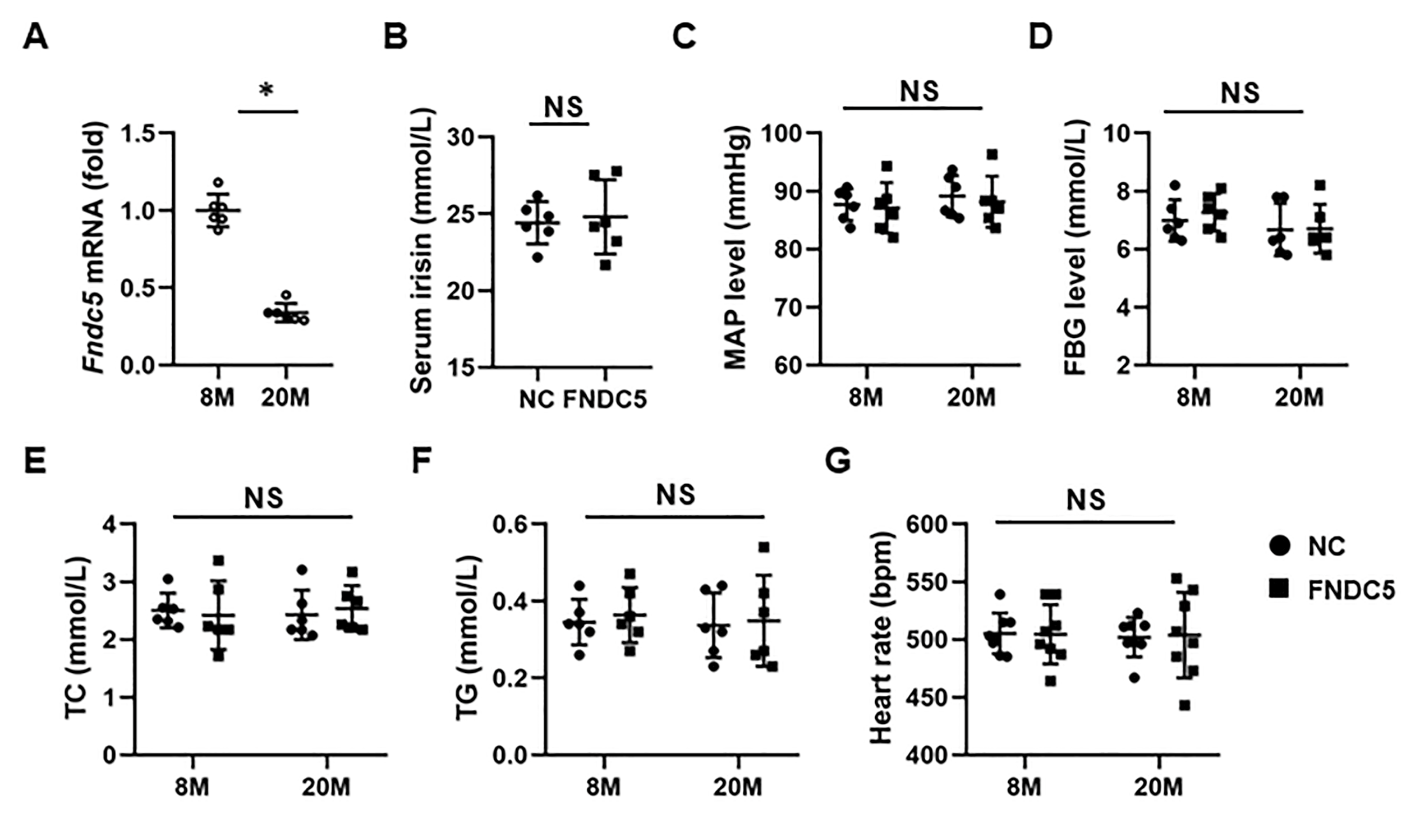


**Figure S1.** **Effects of FNDC5 on systemic metabolism in mice.** (A) The mRNA level of *Fndc5* in 8-(month) M-old young and 20-M-old aging hearts (n=6). (B) Mice were injected with AAV9-FNDC5 (1 × 10^11^ viral genome per mouse) from the tail vein for 8 weeks to specifically overexpress FNDC5 in the myocardium or AAV9-NC as a control, and then the serum irisin level was detected after AAV9-NC or AAV9-FNDC5 injection by an ELISA kit (n=6). (C) 6-M-old young and 18-M-old aging mice were injected with AAV9-FNDC5 for 8 weeks to overexpress FNDC5 or AAV9-NC as a control, and then mean arterial pressure (MAP) was determined in mice among groups (n=6). (D) Fasting blood glucose (FBG) in mice among groups (n=6). (E-F) The serum total cholesterol (TC) and triglyceride (TG) levels among groups (n=6). (G) Heart rate among groups (n=8). Values represent the mean ± standard deviation. **P* < 0.05 versus the matched group. NS indicates no significance.

**
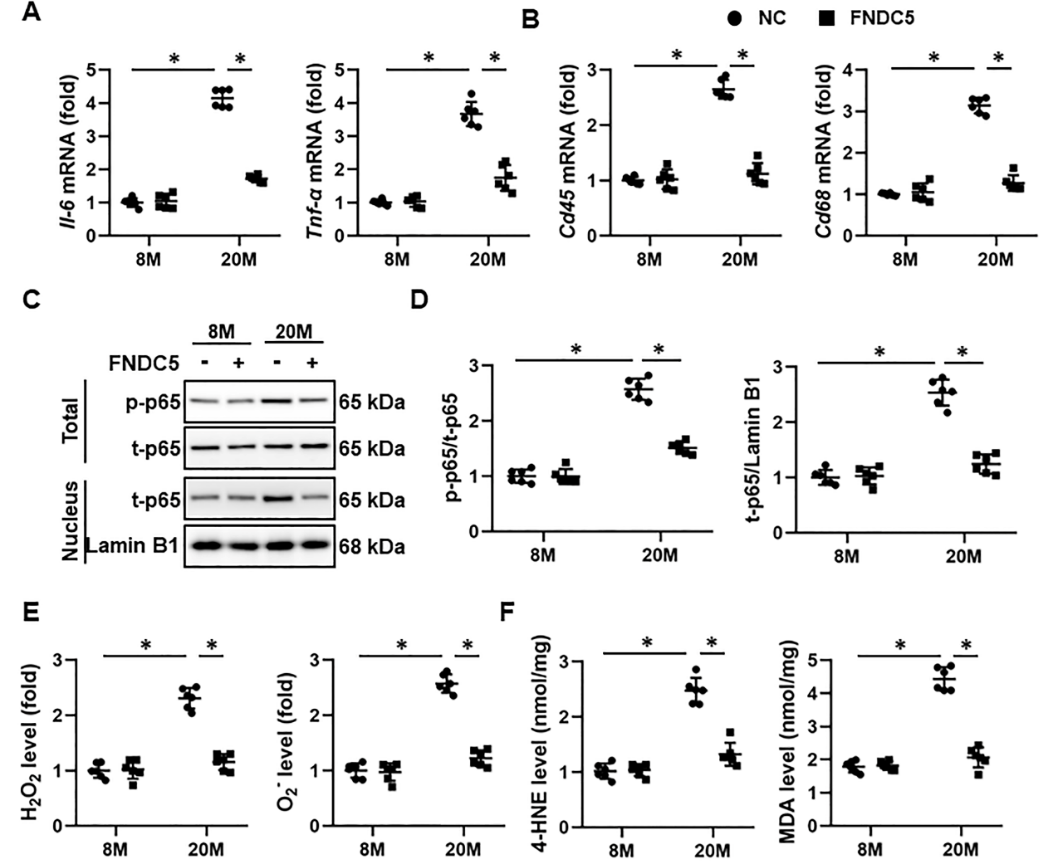
**

**Figure S2.** **FNDC5 alleviates aging-related cardiac inflammation and oxidative damage in mice** (A) 6-month (M)-old young and 18-M-old aging mice were injected with AAV9-FNDC5 (1 × 10^11^ viral genome per mouse) from the tail vein for 8 weeks to specifically overexpress FNDC5 in the myocardium or AAV9-NC as a control, and relative interleukin-6 (*Il-6*) and tumor necrosis factor-α (*Tnf-α*) mRNA levels in the heart were determined (n=6). (B) Relative cluster of differentiation 45 *(Cd45)* and *Cd68* mRNA levels in the heart (n=6). (C-D) Western blot images and the statistical results (n=6). (E) The hydrogen peroxide (H_2_O_2_) production and superoxide anion (O_2_^-^) level in the myocardium (n=6). (F) The 4-hydroxynonenal (4-HNE) and malondialdehyde (MDA) levels in the myocardium (n=6). Values represent the mean ± standard deviation. **P* < 0.05 versus the matched group.

**
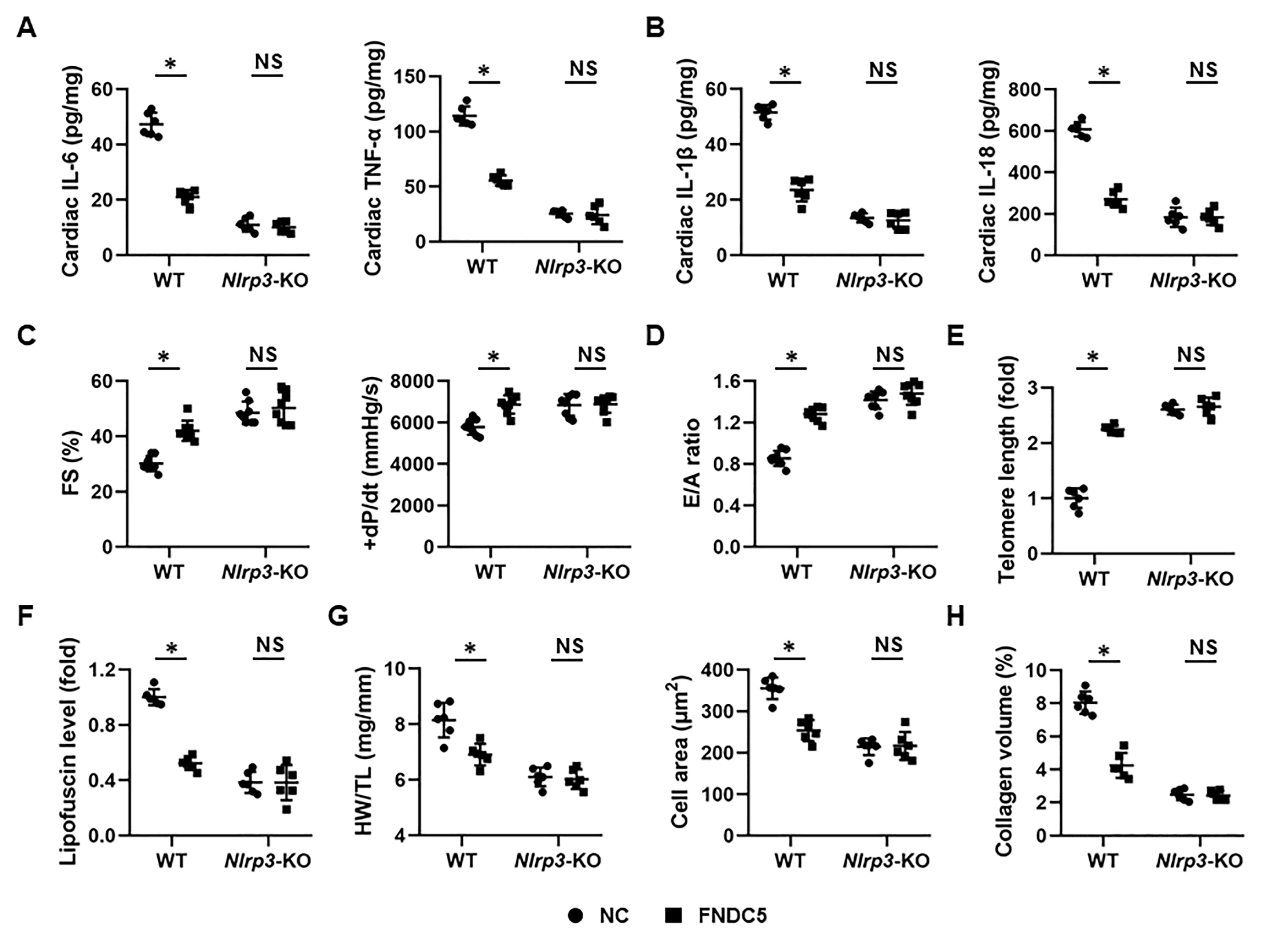
**

**Figure S3.** **NLRP3 inhibition is required for FNDC5-mediated cardioprotective effects in aging mice.** (A) 18-month (M)-old aging *Nlrp3*-knockout (KO) mice or wild type (WT) littermates were injected with AAV9-FNDC5 (1 × 10^11^ viral genome per mouse) from the tail vein for 8 weeks to specifically overexpress FNDC5 in the myocardium or AAV9-NC as a control, and the myocardial interleukin-6 (IL-6) and tumor necrosis factor-α (TNF-α) levels were determined by ELISA kits (n=6). (B) The myocardial IL-1β and IL-18 levels determined by ELISA kits in mice (n=6). (C-D) Echocardiographic and hemodynamic parameters of cardiac function in mice, including fractional shortening (FS), the peak rates of isovolumic pressure development (+dP/dt) in left ventricles and ratio of the early (E) to late (A) ventricular filling velocities (n=8). (E) Relative telomere length in murine hearts (n=6). (F) Cardiac lipofuscin content (n=6). (G) Quantification of the heart weight-to-tibia length (HW/TL) and cardiomyocytes area in mice (n=6). (H) Average collagen volume in mice (n=6). Values represent the mean ± standard deviation. **P*<0.05 versus the matched group. NS indicates no significance.

**
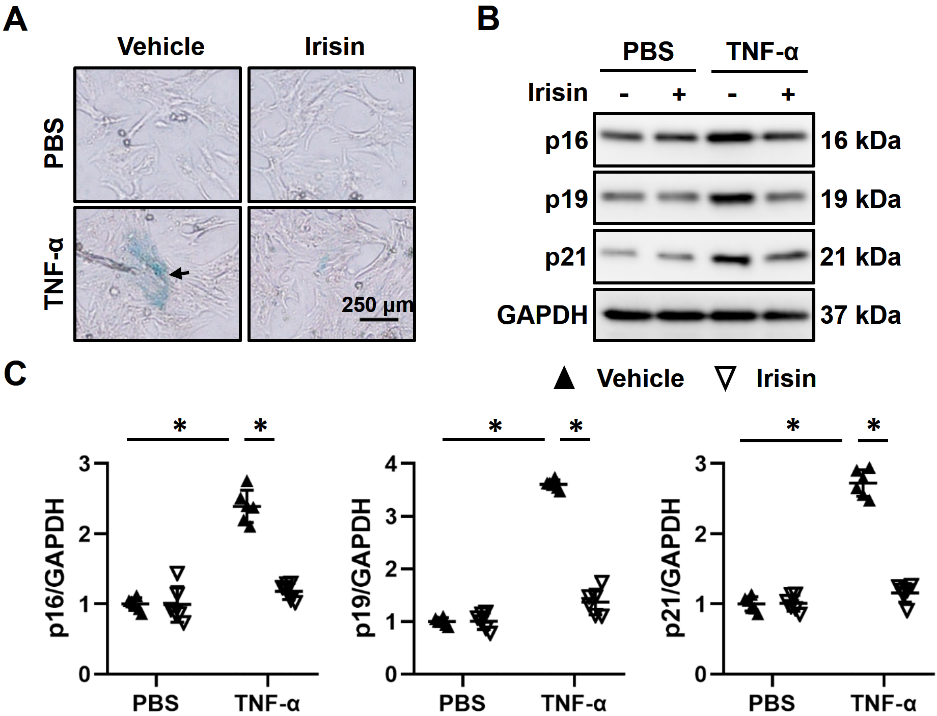
**

**Figure S4.** **Irisin attenuates TNF-α-induced inflammaging *in vitro*.** (A) Neonatal rat cardiomyocytes (NRCMs) were treated with 20 nmol/L irisin for 24 h, followed by the stimulation with 100 ng/mL tumor necrosis factor-α (TNF-α) or phosphate buffered saline (PBS) for an additional 24 h to mimic inflammaging *in vitro*, and the SA β-gal staining images of NRCMs were provided (n=6). (B-C) Western blot images and the statistical results (n=6). Values represent the mean ± standard deviation. **P* < 0.05 versus the matched group.

**
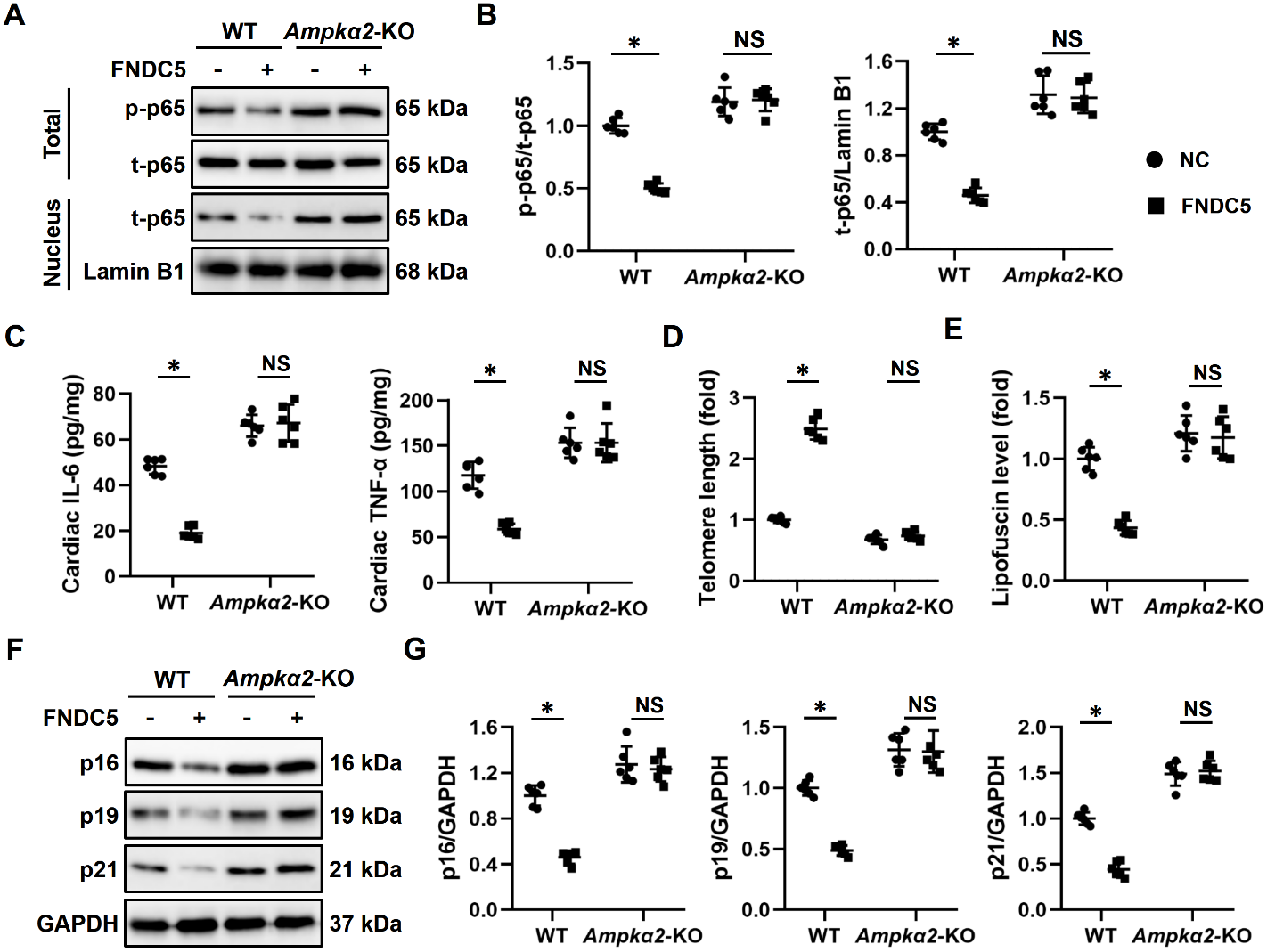
**

**Figure S5.** ***Ampkα* deficiency abrogates FNDC5-mediated anti-inflammatory effect in aging mice.** (A-B) 18-month (M)-old aging *Ampkα2*-(knockout) KO mice or wild type (WT) littermates were injected with AAV9-FNDC5 (1 × 10^11^ viral genome per mouse) from the tail vein for 8 weeks to specifically overexpress FNDC5 in the myocardium or AAV9-NC as a control, and Western blot was performed to detect NF-κB p65 phosphorylation and nuclear translocation (n=6). (C) The myocardial interleukin-6 (IL-6) and tumor necrosis factor-α (TNF-α) levels in mice (n=6). (D) Relative telomere length in murine hearts (n=6). (E) Cardiac lipofuscin content (n=6). (F-G) Western blot images and the statistical results (n=6). Values represent the mean ± standard deviation. **P* < 0.05 versus the matched group. NS indicates no significance.

**
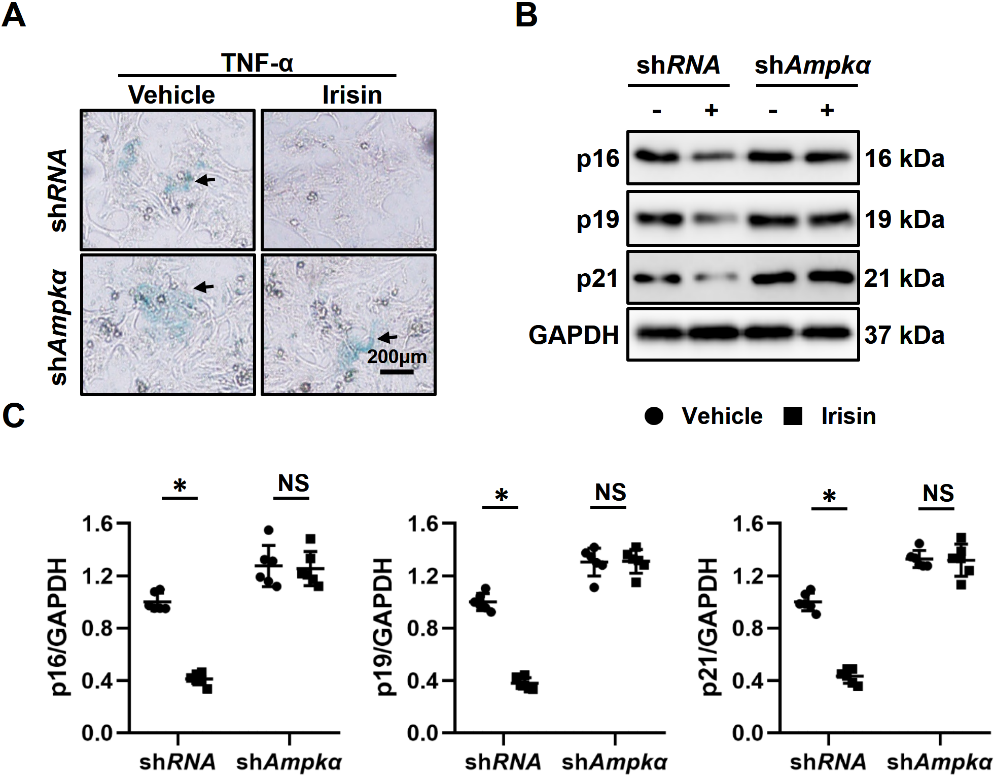
**

**Figure S6.** ***Ampkα* knockdown abolishes the inhibitory effects of irisin on cellular senescence *in vitro*.** (A) Neonatal rat cardiomyocytes (NRCMs) were pre-infected with sh*RNA* or sh*Ampkα* (multiplicity of infection=150) for 4 h and maintained for an additional 24 h, which were then incubated with 20 nmol/L irisin for 24 h, followed by tumor necrosis factor-α (TNF-α, 100 ng/mL) stimulation for an additional 24 h. The SA β-gal staining images of NRCMs were provided (n=6). (B-C) Western blot images and the statistical results (n=6). Values represent the mean ± standard deviation. **P* < 0.05 versus the matched group. NS indicates no significance.

**
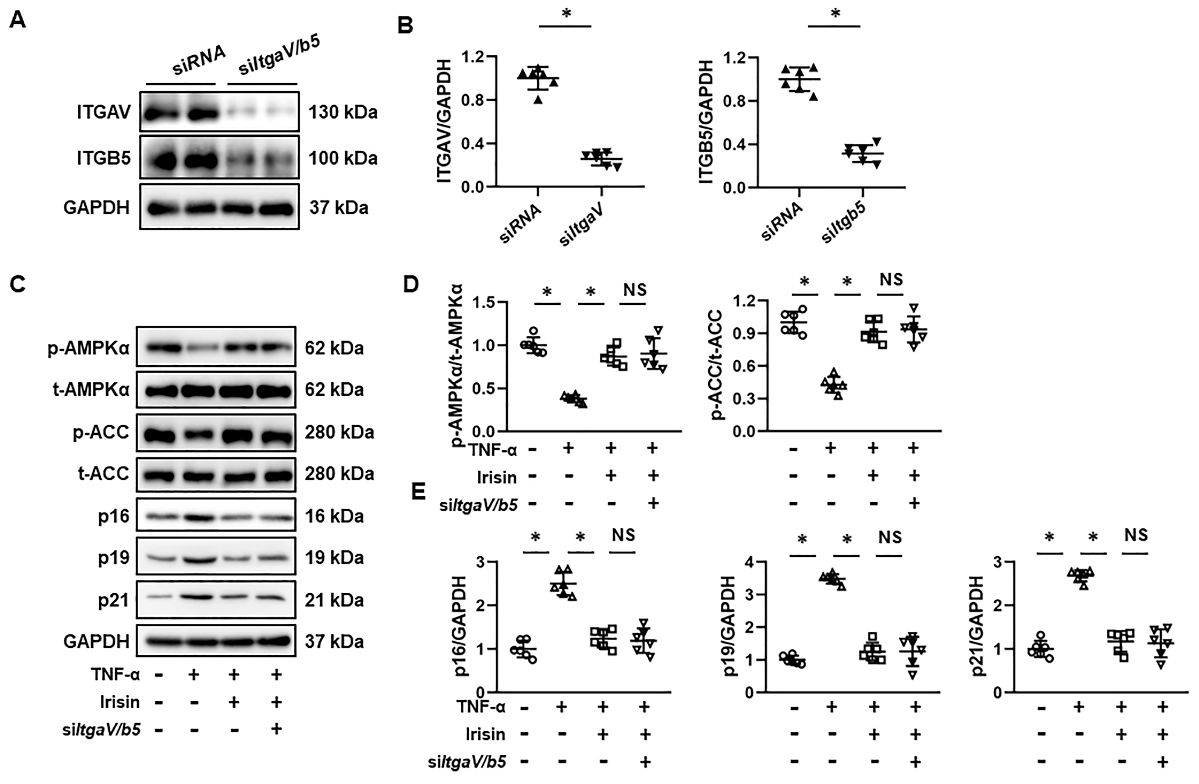
**

**Figure S7. FNDC5 attenuates cardiac aging in an integrin αV/β5-independent manner.** (A-B) Neonatal rat cardiomyocytes (NRCMs) were transfected with 50 nmol/L small interfering RNA against integrin αV and integrin β5 (si*ItgaV/b5*) or scrambled si*RNA*, and then the knockdown efficiency in NRCMs was verified by Western blot (n=6). (C-E) NRCMs were pre-transfected with 50 nmol/L si*ItgaV/b5* for 4h and maintained for an additional 24 h, which were then incubated with 20 nmol/L irisin for 24 h, followed by tumor necrosis factor-α (TNF-α, 100 ng/mL) stimulation for an additional 24 h. Western blot images and statistical results in relative groups were provided (n=6). Values represent the mean ± standard deviation. **P* < 0.05 versus the matched group. NS indicates no significance.

**
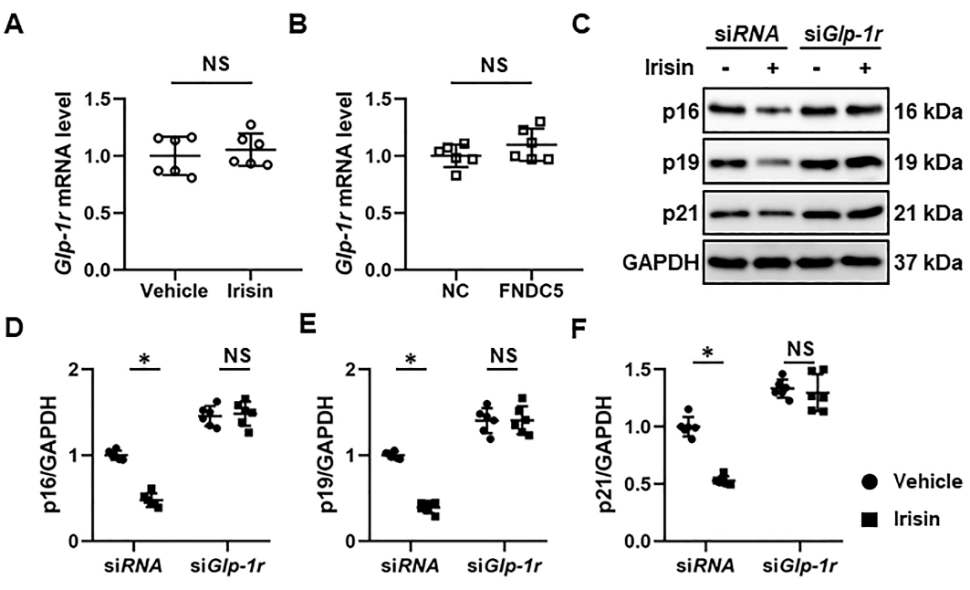
**

**Figure S8. FNDC5 attenuates cardiac aging via blocking the lysosomal degradation of GLP-1R.** (A-B) The mRNA levels of *Glp-1r* in irisin-treated neonatal rat cardiomyocytes (NRCMs) and FNDC5-overexpressed hearts (n=6). (C-F) NRCMs were transfected with si*Glp-1r* (50 nmol/L) for 4 h and maintained for an additional 24 h, which were then incubated with 20 nmol/L irisin for 24 h, followed by tumor necrosis factor-α (100 ng/mL) stimulation for an additional 24 h. Representative images of Western blot and statistical results in NRCMs were provided (n=6). Values represent the mean ± standard deviation. **P* < 0.05 versus the matched group. NS indicates no significance.


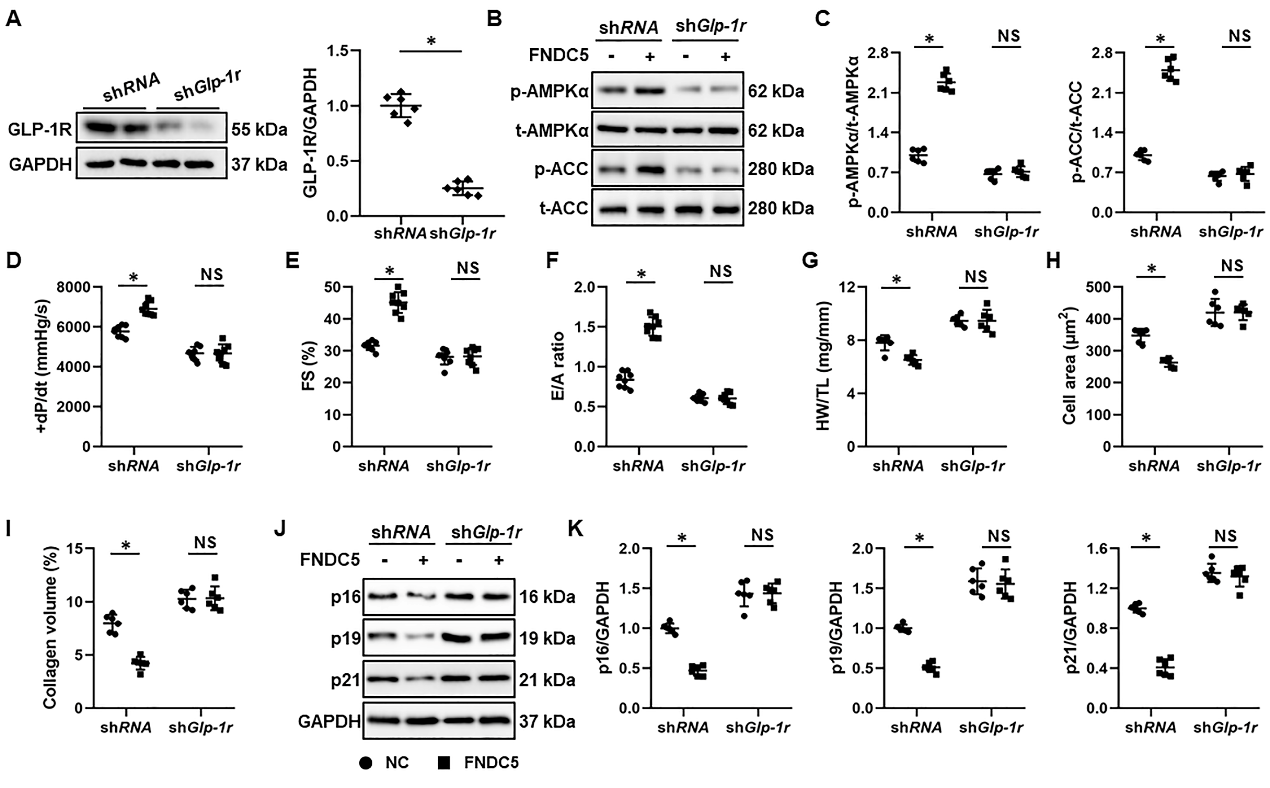


**Figure S9.** ***Glp-1r* knockdown abrogates FNDC5-mediated cardioprotective effect in aging mice.** (A) Mice were intravenously injected with sh*Glp-1r* or sh*RNA* (1 × 10^11^ viral genome per mouse) from the tail vein for 4 weeks, and Western blot was performed to identify the knockdown efficiency in murine hearts (n=6). (B-C) Aging mice were intravenously injected with sh*Glp-1r* or sh*RNA* for 4 weeks, and then received AAV9-FNDC5 injection (1 × 10^11^ viral genome per mouse) from the tail vein for additional 8 weeks to specifically overexpress FNDC5 in the myocardium. Representative Western blot images and statistical results were provided (n=6). (D-F) Hemodynamic and echocardiographic parameters of cardiac function in mice, including the peak rates of isovolumic pressure development (+dP/dt) in left ventricles, fractional shortening (FS) and ratio of the early (E) to late (A) ventricular filling velocities (n=8). (G) Heart weight-to-tibia length (HW/TL) (n=6). (H-I) Quantification of cardiomyocytes area and average collagen volume in mice (n=6). (J-K) Representative Western blot images and statistical results (n=6). Values represent the mean ± standard deviation. **P* < 0.05 versus the matched group. NS indicates no significance.

**
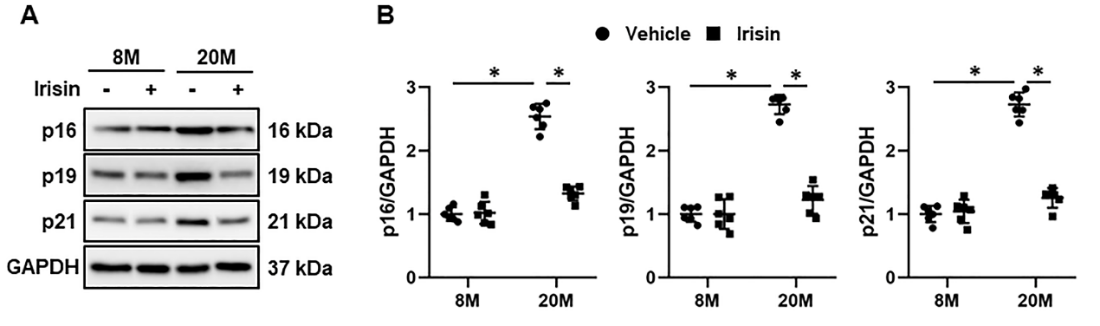
**

**Figure S10. Irisin infusion mitigates cardiac aging in mice.** (A-B) 6-month (M)-old or 18-M-old mice were subcutaneously infused with irisin (12 nmol/kg/day) for 2 M, and Western blot was performed to analyze cellular senescence in murine hearts (n=6). Values represent the mean ± standard deviation. **P* < 0.05 versus the matched group. NS indicates no significance.

**
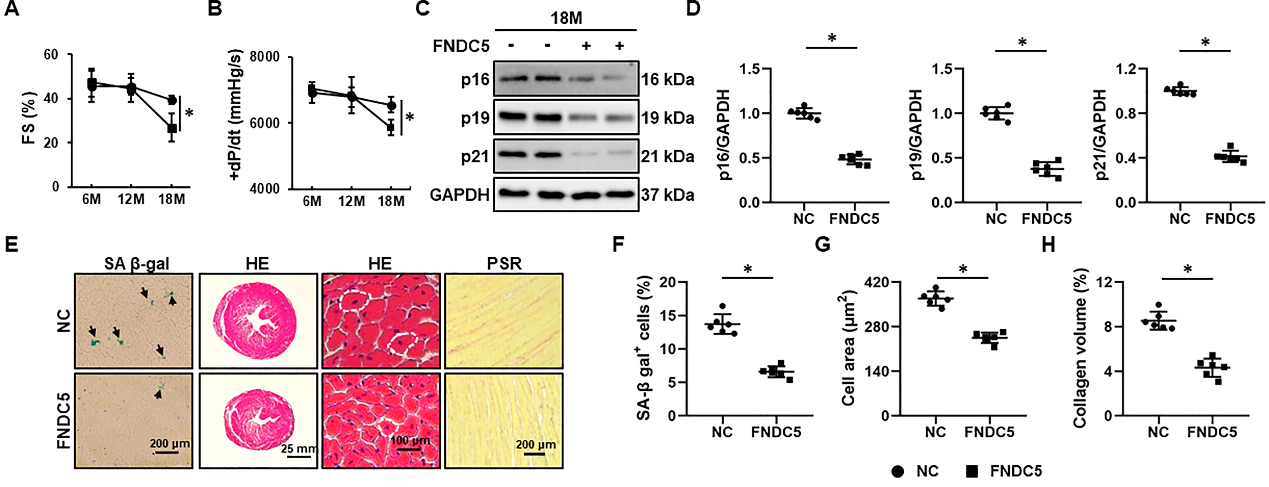
**

**Figure S11. FNDC5 delays the onset of cardiac dysfunction during aging process in mice.** (A-B) 6-month (M)-old mice were injected with AAV9-FNDC5 (1 × 10^11^ viral genome per mouse) or AAV9-NC from the tail vein, and hemodynamic and echocardiographic parameters were analyzed at 12 M after AAV9 injection, including fractional shortening (FS) and the peak rates of isovolumic pressure development (+dP/dt) in left ventricles (n=8). (C-D) Western blot images and statistical results of p16, p19 and p21 (n=6). (E) Representative images of SA β-gal, hematoxylin-eosin (HE) and picric sirius red (PSR) staining in heart (n=6). (F-H) Quantitative data of SA β-gal-positive cells, cardiomyocytes area and average collagen volume in murine hearts (n=6). Values represent the mean ± standard deviation. **P* < 0.05 versus the matched group.
